# A Novel IDO1/NE Dual Inhibitor, IMM‐H018 Prevents the Primary and Secondary Sepsis and Ameliorates the Kidney Injury Through Inhibiting the Cytokine Storm and Microthrombosis, and Reversing Immunosuppression

**DOI:** 10.1002/advs.76504

**Published:** 2026-07-20

**Authors:** Yi Zhou, Xiaodi Zhao, Xiaochen Pan, Zhiling Ma, Huaqing Cui, Hui Wen, Peng Wang, Xiufeng Liao, Kejie Qin, Yuchen Wang, Li Sheng, Sen Zhang

**Affiliations:** ^1^ State Key Laboratory of Bioactive Substances and Functions of Natural Medicines Institute of Materia Medica Chinese Academy of Medical Sciences & Peking Union Medical College Beijing P. R. China; ^2^ Beijing Easyresearch Technology Limited Beijing P. R. China; ^3^ Blood Transfusion Department Lishui City People's Hospital Zhejiang Province P. R. China

**Keywords:** Acute kidney injury, dual inhibitor, IDO1, immune suppression, NE, Sepsis

## Abstract

Sepsis is characterized by the simultaneous presence of hyperinflammation, immunosuppression, and coagulation abnormalities, making single‐target therapies insufficient. In this study, we explored a strategy that concurrently suppresses neutrophil‐mediated inflammation and reverses immune dysfunction. Combined treatment with the neutrophil elastase (NE) inhibitor Sivelestat and the indoleamine 2,3‐dioxygenase‐1 (IDO1) inhibitor Epacadostat showed superior efficacy to either monotherapy in LPS‐ and CLP‐induced septic mice. Based on this concept, a novel dual IDO1/NE inhibitor, IMM‐H018, was designed and synthesized. IMM‐H018 effectively inhibited both IDO1 and NE activities in vitro and in vivo and significantly improved survival in CLP‐induced sepsis. In low‐dose LPS models, IMM‐H018 reduced systemic inflammation and restored immune function in peripheral blood, thymus, and spleen, outperforming the combination therapy. Furthermore, IMM‐H018 alleviated sepsis‐associated acute kidney injury by improving renal perfusion and reducing microthrombosis through inhibition of the IDO1‐Kyn‐AhR‐TF pathway. In a two‐hit sepsis model, IMM‐H018 prevented secondary infection, enhanced bacterial clearance through improved phagocytosis, protected against renal damage, and delayed progression from acute kidney injury to chronic kidney dysfunction. These findings identify IMM‐H018 as a promising therapeutic candidate for sepsis and sepsis‐associated kidney injury.

## Graphical abstract

IMM‐H018, a dual IDO1/NE inhibitor, demonstrates potent therapeutic efficacy in sepsis by simultaneously targeting excessive inflammation and immune dysfunction. It prevents both primary and secondary sepsis through anti‐inflammatory, immune‐restoring, and renoprotective mechanisms, reducing organ damage, improving immune homeostasis, preserving kidney function, and ultimately enhancing survival outcomes in preclinical models.

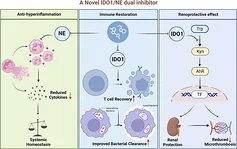

## 1 Introduction

Sepsis is defined as life‐threatening organ dysfunction that arises from a disordered immune response to infection. It encompasses a multifaceted process of hyperinflammation, immune suppression and coagulation abnormalities, resulting in tissue damage and organ injury with high mortality [1, 2]. At first stage, during the hyperinflammatory phase, immune cells release pro‐inflammatory cytokines, leading to hypotension caused by vasodilation, endothelial injury, coagulation activation, and complement system activation, which further amplifies inflammation and tissue damage [3]. However, soon, the body shifts to an immunosuppressive phase, mostly due to lymphocyte apoptosis, which not only is directly associated with the patient survival but also increases susceptibility to secondary infections [4, 5]. During the whole process, coagulation abnormality is an important mechanism for organ injuries, and tissue factor (TF) activation, thrombin generation and microvascular thrombosis contribute to this condition [6, 7].

Sepsis‐associated acute kidney injury (SA‐AKI) presents a common and severe complications of sepsis, resulting in worsened clinical outcomes, prolonged hospitalization, and markedly increased mortality [8]. The pathogenesis of SA‐AKI is multifactorial, with renal vascular dysfunction playing a central role [8]. Studies in large animal models, both clinical and experimental, have revealed that elevated renal vascular resistance contributes to reduced renal perfusion during sepsis, driven in part by microthrombosis and the persistent accumulation of neutrophil extracellular traps (NETs) in the renal vasculature [9]. This microcirculatory impairment results in regional ischemia and hypoxia, disrupting glomerular filtration and triggering tubular epithelial cell injury and demise, ultimately resulting in renal impairment [10]. The combination of hypoperfusion, inflammation, and direct cellular damage exacerbates kidney injury, making SA‐AKI a significant determinant of poor prognosis in septic patients.

Current sepsis/SA‐AKI management relies on early antibiotics, hemodynamic support, and renal replacement therapy. Emerging therapies target immune dysregulation, microthrombi, and cellular injury [11], with recombinant human alkaline phosphatase (recAP, sevuparin) [12], IL‐6 inhibitors [13], and NETs inhibitors [14] showing promise. However, clinical trials of candidate drugs for sepsis have yielded unsatisfactory results, highlighting the urgent need for novel therapeutic strategies. Given the complex pathophysiology of sepsis/SA‐AKI, which involves multiple targets and pathways, combination therapy has garnered increasing attention as a means to improve treatment efficacy [15].

Neutrophil elastase (NE), a serine protease secreted via activated neutrophils, exerts dual effects in sepsis and SA‐AKI [16]. While critical for microbial elimination, excessive NE activity worsens tissue damage by promoting inflammation, degrading extracellular matrix components, and disrupting endothelial and epithelial barriers [9]. In sepsis, NE amplifies systemic inflammation by processing cytokines, enhancing vascular permeability, and contributing to NET formation, which can lead to microvascular thrombosis [9]. In sepsis‐induced AKI, NE‐mediated endothelial injury impairs renal perfusion, while direct tubular damage occurs through degradation of adhesion molecules and induction of apoptosis [17]. Additionally, NE activates pro‐fibrotic pathways, worsening renal injury and induces AKI to CKD transition. NE inhibitors, such as Sivelestat (Siv), are not currently first‐line treatments for sepsis or sepsis‐induced acute kidney injury (AKI) in global clinical practice, though they are approved in Japan and South Korea for acute lung injury (ALI/ARDS) associated with systemic inflammation, but large‐scale trials like the STRIVE study have not demonstrated a clear mortality benefit in sepsis [18]. However, their potential role in specific subgroups‐such as patients with excessive neutrophil activation or NETosis warrants further research, particularly in sepsis‐induced AKI where NE contributes to tubular and glomerular dysfunction [19, 20, 21].

Although NE inhibitor could reduce the Nets mediated inflammation in sepsis, but it is hard to reverse the immunosuppression during the sepsis, which is more important for patient prognosis. Increasing evidence points to a therapeutic benefit of immunomodulatory treatment for sepsis, especially the application of immune check point inhibitors [22]. Previous studies have demonstrated that blocking PD‐1/PD‐L1 or CTLA4 by monoclonal antibodies could improve survival in experimental sepsis via correcting the monocyte dysfunction and suppressing lymphocyte apoptosis [23, 24].

Indoleamine 2,3‐dioxygenase‐1 (IDO1), another endogenous immune checkpoint enzyme, which catalyze the tryptophan into the kynurenine, is significantly upregulated in the sepsis, both in circulation or kidneys [25, 26]. IDO1‐dependent tryptophan catabolism ultimately leads to extensive immunosuppression under both physiological and pathophysiological states, including sepsis or cancer immune escape [27]. IDO1 activity is strongly linked to sepsis‐induced immune dysfunction/immunosuppression, particularly during the tolerant phase. Elevated Kyn/Trp ratio is found in non‐survivors from sepsis relative to survivors, and the IDO1 activity and plasma concentration is directly and positively correlated with lethal rate of patients [28]. In the AKI patients, the elevated urinary excretions of kynurenine and kynurenic acid, two significant inflammatory metabolites derived from tryptophan degradation, correlate with worse renal function and poorer overall prognosis [28]. Joshua A.Waler also has reported that IDO1‐Kyn‐tissue factor (TF) axis contribute to the vascular thrombosis in the chronic kidney dysfunctional patients [29], which is also an import pathological mechanism to SA‐AKI. Prior to sepsis induction, blocking IDO1 activity via either gene knockout or administration of the IDO1 inhibitors 1‐methyl‐D‐tryptophan (1‐MT) or Epacadostat (Epa) significantly decreases septic mouse mortality, and this intervention also preserves organ function [30, 31]. Compared with PD‐1/PD‐L1 and CTLA4, enzyme characteristics of IDO1 are more suitable for designing small molecular inhibitors. Although most IDO1 inhibitors, such as Epacadostat, fail to Phase III clinical trial for solid cancers, but it can be suitable for drug candidate to treat sepsis, also as small molecules, it has several advantages than monoclonal antibodies. Therefore, based on that, we hypothesize that a combination of NE inhibitor and IDO1 inhibitor not only reduces the hyperinflammation in the initial phase of sepsis, but also restore the comprised immune activity in the middle or later stage of sepsis, and is more efficacious than monotherapy for alleviating sepsis and SA‐AKI in vitro and in vivo.

To better understand the scope of this conceptual combined therapeutic strategy and expand the combinatorial chemistry to develop novel dual IDO1/NE inhibitor candidates, here, we first demonstrate that combining the Epa with Siv presents a synergistically beneficial therapeutic strategy against sepsis and SA‐AKI in mice. We observed that combining Epa and Siv exhibits superior efficacy than either agent alone for the prolonged survival, reduced lymphocyte apoptosis and thrombosis, restoring the renal function in septic mice. All the data suggested that combining Epa and Siv presents a viable alternative therapeutic modality. After the hypothesis has been confirmed, we used AI tool to design a series of novel compounds, which have both inhibitory activity on NE and IDO1, and one of them, named IMM‐H018, has showed potential to be a novel drug candidate against sepsis and SA‐AKI by in vitro and in vivo screening.

## 2 Materials and Methods

### 2.1 Animals

Male C57BL/6J mice, weighing 18–20 g, age 6–8 weeks, were obtained from the SPF biotechnology (Beijing, China). Mice were housed in specific pathogen‐free (SPF) facilities and acclimated for at least three days prior to experiments. All animal procedures were approved by the Beijing Municipal Ethics Committee for Laboratory Animals (approval No. IMM‐N‐25‐0504) and conducted in compliance with the Chinese guidelines for laboratory animal welfare and ethics.

### 2.2 Cecal Ligation‐Puncture and LPS High Lethality Sepsis Survival Analysis

Polymicrobial sepsis was induced by cecal ligation‐puncture (CLP) as previously reported [23]. Following anesthesia with isoflurane, a midline abdominal incision was performed to expose the cecum. A 7.5 mm segment of the cecum was ligated from the tip toward the ileocecal valve. The ligated cecum was then perforated twice by an 18‐gauge needle, and a small amount of fecal material was gently extruded. The cecum was returned to the abdominal cavity, and the abdominal incision was subsequently sutured. Following surgery, mice received 1 mL of sterile saline for fluid resuscitation and maintained free access to water and food both before and after the procedure.

Survival was also evaluated in a lethal LPS‐induced endotoxemia model. LPS from Escherichia coli 055: B5 (Sigma‐Aldrich, L2880) was freshly prepared in sterile 0.9% saline prior to use. Mice were intraperitoneally injected with LPS at a dose of 30 mg/kg, and after that, the animals were monitored for 96 h, and the time of death was recorded for each mouse to calculate survival rates.

Each experiment contained five groups, which were control, vehicle‐treated model, Epacadostat (Epa, 80mg/kg), Sivelestat (Siv, 40mg/kg), and Epa (80mg/kg) + Siv(40mg/kg) respectively. The determination of drug dosage was referred from several publications combined preliminary study in our laboratory [32, 33, 34]. Different drugs were administrated (i.p.) two days earlier before sepsis induction, and continued once per day until the termination, and survival rates were recorded. The detailed experimental design is presented in Figure 1C.

**FIGURE 1:**
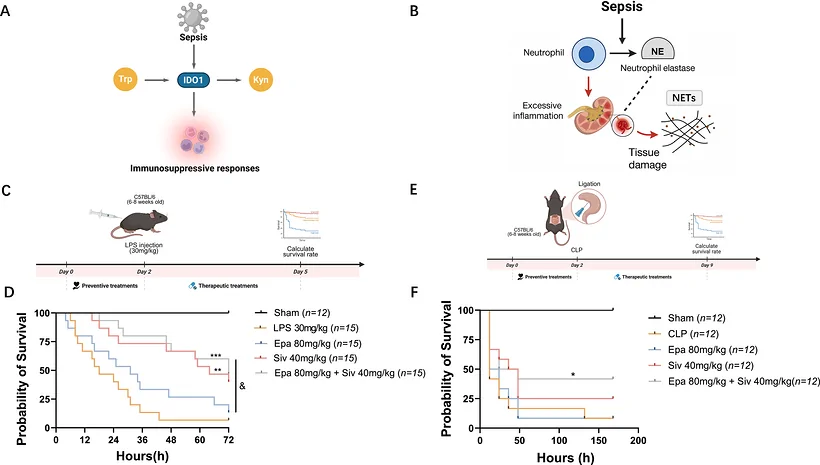
Combination of Epa and Siv prolongs the survival of experimental sepsis better than treatment alone. (A) Sepsis induces indoleamine 2,3‐dioxygenase 1(IDO1) activity, converting tryptophan (Trp) to kynurenine (Kyn) to drive immunosuppression. (B) Neutrophil elastase (NE) release in sepsis promotes excessive inflammation, neutrophil extracellular trap (NET) formation, and tissue damage. (C) Experimental design for the LPS‐induced sepsis model: C57BL/6J mice (6‐8 weeks) received LPS (30 mg/kg), with treatments administered as indicated. Survival was observed for 3 days. D. Kaplan‐Meier survival curves for the LPS model: Sham (*n* = 12), LPS (*n* = 15), Epa 80 mg/kg (*n* = 15), Siv 40 mg/kg (*n* = 15), and Epa + Siv (*n* = 15). Log‐rank test: **p < 0.01, ***p < 0.001 vs. LPS. ^&^ *p < 0.05* vs Epa +Siv. E. Experimental design for the CLP‐induced sepsis model: C57BL/6J mice (6‐8 weeks) underwent cecal ligation‐puncture (CLP), with treatments administered as indicated. Survival was observed for 7 days. F. Kaplan‐Meier survival curves for the CLP model: Sham (*n* = 12), CLP (*n* = 12), Epa 80 mg/kg (*n* = 12), Siv 40 mg/kg (*n* = 12), and Epa + Siv (*n* = 12). Log‐rank test: *p < 0.05 vs. CLP.

### 2.3 Low‐Lethality Murine Sepsis Model and Two‐Hit Sepsis Model

Non‐lethal sepsis was established in mice by administering a low dose of LPS (10mg/kg, *i.p*.) Saline, Epa (80mg/kg) Siv (40mg/kg), and the combination were pre‐treated twice, two days earlier, before sepsis induction (*N* = 6 per group). Half an hour later LPS injection, the different drugs were administrated for the third time and the detailed procedure was illustrated in the Figure 2A. 24 h later, 20 uL peripheral plasma was harvested from the posterior canthus in heparin pretreated tubes for complete blood count analysis using a Celltac E MEK‐7222 analyzer (Nihon Kohden, Japan). The remaining blood was harvested in untreated tubes, with serum subsequently isolated for cytokine analysis. Following euthanasia by cervical dislocation, major organs were harvested from the animals and weighed for determination of relative organ weight. The kidneys were formalin‐fixed and paraffin‐embedded for pathological examination.

**FIGURE 2:**
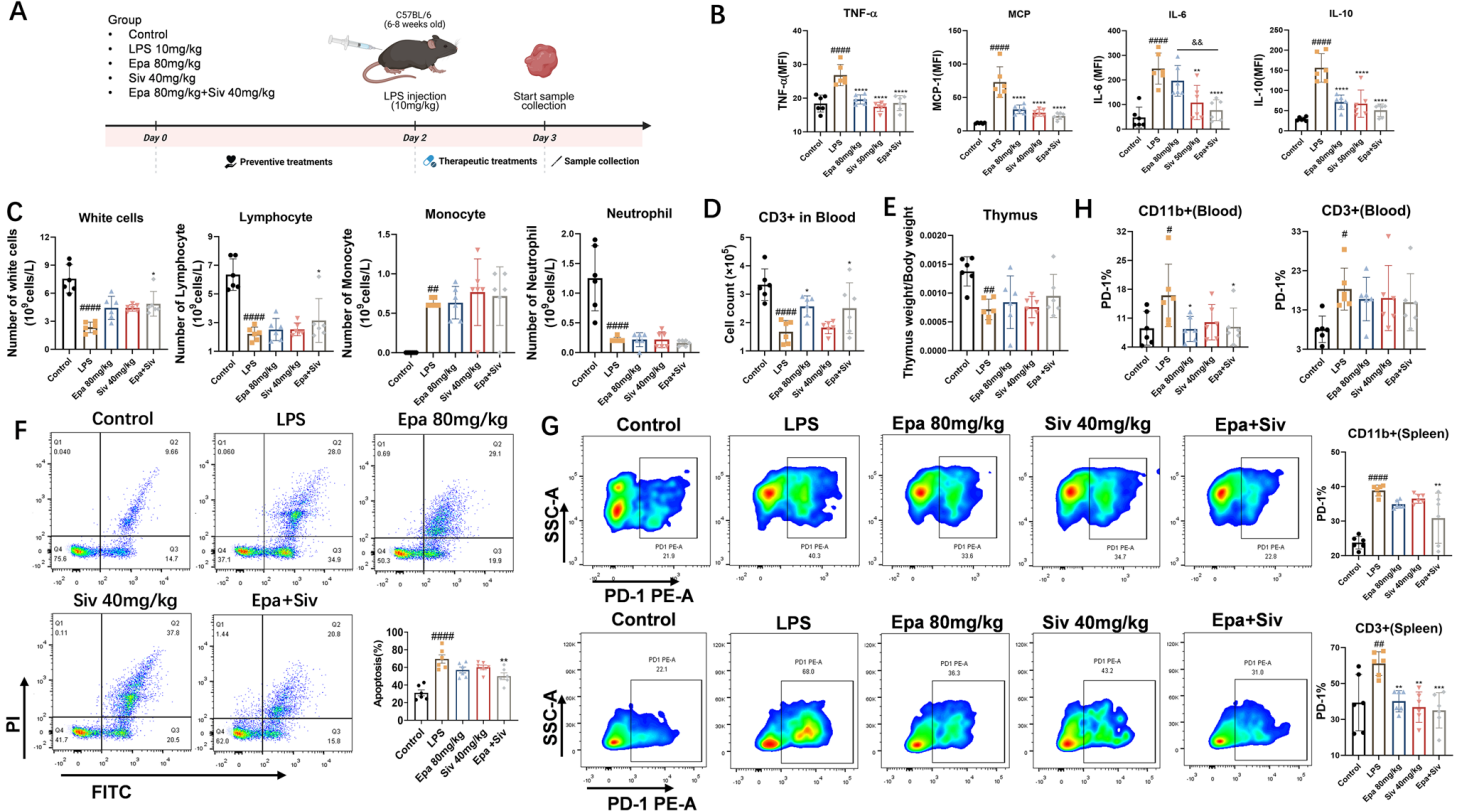
Epacadostat plus Sivelestat improves LPS‐induced inflammation and immunosuppression in mice. (A) Experimental scheme of LPS‐induced sepsis and drug treatments. (B) Serum levels of cytokines including TNF‑α, MCP‑1, IL‐6, and IL‑10 in mice from different groups. (C) Peripheral blood counts of lymphocytes, monocytes, and neutrophils in control, LPS, and treated mice. (D) Flow cytometric analysis of circulating CD3^+^ T lymphocytes in the blood. (E) Relative thymus weight in mice from each group. (F) Apoptosis of CD3^+^ T lymphocytes in the thymus analyzed by flow cytometry. (G, H) Flow cytometric quantification of PD‑1^+^ cell proportions in CD3^+^ T cells and CD11b^+^ monocytes in the blood and spleen. Data are presented as mean ± SD. *p < 0.05, **p < 0.01, ***p < 0.001, ****p < 0.0001 vs. LPS group. *^#^p < 0.05, ^##^p < 0.01, ^###^p < 0.001, ^####^p < 0.0001* vs. Control group. *^&&^p < 0.01* vs. Epa + Siv group.

A simple “two‐hit” sepsis model was established. Briefly, mice were first subjected to an initial insult via intraperitoneal injection of LPS (10 mg/kg) to induce an immunosuppressive state. Three days later, during the maintenance of immunosuppression, mice were challenged with a second hit by intraperitoneal injection of Escherichia coli ATCC 25922 (2 × 10^7^ CFU/mouse) to mimic secondary bacterial infection. Each group consisted of 8 mice. 24 h after bacterial challenge, serum was harvested for bacterium burden analysis. Briefly, Anticoagulated whole blood was diluted 100‐fold with sterile saline. Each sample (100 µL) from different groups was evenly distributed onto bacterial culture plates, followed by overnight incubation at 37°C. Colony‐forming units (CFUs) were subsequently measured for bacterial burden determination. One week later, major organs following euthanasia were collected for histopathological analysis. The detailed procedure was shown in Figure 8A.

### 2.4 Biochemical Assays

The concentrations of cytokines (TNF‐α, MCP‐1, IL‐6, IL‐10) were assayed by a multi‐cytokine assay kit (Cat. No. 740251, LEGENDplex, BioLegend, USA). Interleukin 6 (IL‐6) was detected by Mouse Interleukin 6 (IL‐6) ELISA Kit (JL20268, Jonln, China). Blood urea nitrogen (BUN) was detected by Urea Assay Kit (C013‐2‐1, Nanjing Jiancheng Bioengineering Institute, China). Serum β2‐microglobulin (β2‐MG) and neutrophil gelatinase‐associated lipocalin (NGAL) were detected by enzyme‐linked immunosorbent assay (ELISA) kits (ml037958 for β2‐MG, ml002141 for NGAL, Shanghai Enzyme‐linked Biotechnology Co., Ltd., China). Blood glutamic‐pyruvic transaminase (GPT/ALT) and micro glutamic‐oxalacetic transaminase (GOT/AST) activities were determined using commercially available assay kits (BC1555 for ALT, BC1565 for AST, Solarbio, China).

### 2.5 Determination of Lymphocyte Apoptosis in Thymus

Following LPS challenge (10 mg/kg), the thymuses from septic mic were collected at 24 h. Mouse thymuses were grinded using 1ml PBS. After grinding, pass it through a 40 mesh sieve. Collected the liquid containing cell suspension after grinding and centrifuge at 350g for 5 min, and discarded the supernatant and added 1 ml of red blood cell lysis buffer on ice for 20 min. Centrifuged again at 350g for 5 min, and washed once with PBS to prepare a lymphocyte suspension. The cell suspensions were adjusted to 100 µL and incubated with anti‐mouse CD16/CD32 antibody (Fc receptor blocking antibody) on ice for 15 min, followed by staining with APC‐conjugated anti‐CD3 antibody on ice for 45 min in the dark. After washing with PBS, according to the apoptosis assay kit, 500 µL Banding Buffer was first added to resuspended cells in EP tubes. Added 10 µL PI staining solution and 5 µL FITC staining solution to each EP tube, and incubated in the dark at room temperature for 5 min, and monitored the apoptosis percentage of thymic T cells using flow cytometry (BD Verse).

### 2.6 PD‐1 Expression in Blood and Splenic T Cells and Monocytes

Peripheral blood together with spleen tissues were collected 24 h after LPS (10mg/kg) challenge. Following red blood cell lysis using lysing solution (R1010‐500 mL, Solarbio), the remaining cells were incubated with anti‐CD3, anti‐CD11b or anti‐PD‐1. Data acquisition was carried out on a flow cytometer (BD Verse), and subsequent analyses were performed by FlowJo software (version 10.8.1, Tree Star). The following antibodies were obtained from BioLegend (San Jose, CA, USA): CD3‐APC (480100), CD11b‐APC (480110), and PD‐1‐PE (135205).

### 2.7 Kidney Histopathology and Immunohistochemistry

Kidney tissues were harvested and fixed in 4% paraformaldehyde, followed by routine paraffin embedding. Paraffin blocks were sectioned at 4 µm thickness, and the sections were subsequently subjected to deparaffinization and rehydration prior to hematoxylin and eosin (H&E) staining. Renal injury was assessed by examining multiple pathological features, including glomerular alterations, tubular epithelial vacuolization and dilation, disruption of the proximal tubular brush border, detachment or necrosis of tubular cells, as well as infiltration of neutrophils. The extent of each parameter was semi‐quantitatively scored on a scale ranging from 0 to 4 according to severity based on previous publications [35, 36].

Immunohistochemical analysis was conducted on paraffin‐embedded kidney sections [37, 38]. After deparaffinization and rehydration, antigen retrieval was performed using sodium citrate buffer (pH 6.0). The sections were then washed and incubated overnight at 4°C with primary antibodies against NGAL (1:100, sc‐515876, Santa Cruz), fibronectin (1:100, #62601, CST), fibrinogen alpha chain (1:500, 20645‐1‐AP, Proteintech), and citrullinated histone H3 (CitH3, 1:800, 97272T, CST). Following primary antibody incubation, sections were treated with a horseradish peroxidase‐conjugated secondary antibody (ZSGB‐Bio, Beijing, China) and visualized, with hematoxylin applied for nuclear counterstaining. Staining intensity was quantified using ImageJ software to evaluate the expression levels of the indicated proteins.

### 2.8 Determination of IDO1 Inhibition Activity in A172 Cell‐Based IDO1/Kynurenine Assay

A172 cells (ATCC, MA. USA) were seeded in 96‐well plates at a density of 4 × 10^4^ cells per well in 100 µL culture medium. After 24 h, the medium was replaced with complete medium containing 10% fetal bovine serum supplemented with the test compounds, interferon‐γ (IFN‐γ, final concentration 100 ng/mL), and L‐tryptophan (Trp, final concentration 1 mmol/L). The cells were incubated for an additional 24 h. Subsequently, 100 µL of culture supernatant was transferred to a new 96‐well plate and mixed with 20 uL of 30% (w/v) trichloroacetic acid (TCA) to terminate the reaction and precipitate proteins. The plate was then incubated in a 50°C water bath for 30 min to convert N‐formylkynurenine to kynurenine (Kyn). After cooling to room temperature, an equal volume of 2% (w/v) p‐dimethylaminobenzaldehyde (Ehrlich's reagent) dissolved in acetic acid was added and mixed thoroughly. Following incubation for 5 min, the absorbance was measured at 490 nm using a microplate reader.

### 2.9 Neutrophil Elastase Activity Assay

Neutrophil Elastase Activity was quantified by the commercial kit (ab204730, Abcam). Briefly, the assay was performed in black 96‐well plates. Test compounds at various concentrations were incubated with neutrophil elastase in assay buffer at room temperature for 10 min. Subsequently, the fluorogenic substrate solution was added to initiate the enzymatic reaction. The mixture was incubated at 37°C, and fluorescence intensity was monitored using a microplate reader at an excitation wavelength of 400 nm and an emission wavelength of 505 nm. Enzyme activity in the presence of inhibitors was calculated relative to the control group without inhibitor. The half‐maximal inhibitory concentration (IC_50_) values were determined by nonlinear regression analysis using GraphPad Prism software.

### 2.10 Study of the Interaction Mode of IMM‐H018 Toward IDO1

For in vitro IDO1 enzymatic assay, *D‐tryptophan* was employed as the substrate to minimize substrate inhibition. The reversibility of IMM‐H018 to IDO1 was determined as follow. *D‐tryptophan* (300 µM) was employed as the assay substrate. 0 or 1 µM of IMM‐H018 were incubated with various concentrations (0.1, 0.2, 0.3, 0.4 µM) of IDO1 for 60 min before measuring the production of kynurenine. The plot of reaction rate versus IDO1 concentration was drafted to determine the inhibition mode of IMM‐H018 toward IDO1. The Lineweaver‐Burk plot of IDO1 versus IMM‐H018 were measured following the protocol. Various concentrations (200, 400, 600, 800, 1000, 1200 µM) of *D*‐tryptophan were set as the substrates for each enzymatic assay. 0, 0.25, 0.50, 0.75, 2.50 µM IMM‐H018 were applied to the assay system and incubated for 60 min before ending the reaction. The reaction rates of each IDO1 enzymatic assay were calculated to draw the plot. The UV–vis spectra analysis was carried out as follows. The assay was performed in 100 mM Tris‐HCl (pH 7.4), the concentration of IDO1 was 25 µM. IDO1 in the ferric state was incubated with IMM‐H018 (200 µM) or an equal volume of DMSO for subsequent analysis. UV–vis spectra were collected at the time points of 0, 30, and 60 min from 380 to 500 nm with a slit width of 1 nm. The ferrous state of IDO1 was obtained by incubating the enzyme (25 µM) with 5 mM sodium dithionite in 100 mM Tris‐HCl buffer (pH 7.4) under an argon atmosphere. Thereafter, IMM‐H018 (200 µM) or an equal volume of DMSO was introduced into the IDO1 solution. UV–vis spectra were recorded over the range of 380–500 nm at 0, 30, and 60 min with a slit width of 1 nm. Absorbance was measured using a BioTek Synergy H microplate reader. Data were produced using GraphPad Prism 10.1.2.

### 2.11 Study of the Interaction Mode of IMM‐H018 Toward NE

The reversibility of IMM‐H018 to NE was determined as follows. 50 µM MeOsuc‐AAPV‐AFC was used as the substrate for the assay. 0 or 20 µM of IMM‐H018 were incubated with various concentrations (0.1, 0.2, 0.3, 0.4 µm) of NE. The fluorescence intensity was monitored (380 nm / 460 nm) in real time. The plot of reaction rate versus NE concentration was drafted to determine the inhibition mode of IMM‐H018 toward NE. The Lineweaver‐Burk plot of NE versus IMM‐H018 were measured following the protocol. In this assay, each enzymatic reaction system contained a range of substrate concentrations (40, 80, 120, 160 µm) of MeOsuc‐AAPV‐AFC and was supplemented with 0, 5, or 10 µm of IMM‐H018, respectively. The reaction rates of each NE enzymatic assay were calculated to draw the plot.

### 2.12 Intravital Microscopy for Assessment of Renal Microcirculation

Anticoagulated whole blood was diluted and centrifuged at 1000 rpm for 5 min. Following centrifugation, the erythrocyte fraction was collected, counted, and resuspended to 1 × 10^6^ cells/mL. The cells were labeled with DiD (2.5 µM) and incubated at 37°C for 20 min. After labeling, erythrocytes were resuspended to 2 × 10^8^ cells/mL and kept on ice protected from light until use.

Mice were anesthetized with isoflurane. A 200 µL suspension of DiD‐labeled RBCs was injected via the tail vein, immediately followed by an injection of 200 µL of FITC‐Dextran (8 mg/mL) to visualize the plasma. A small incision was made in the right dorsal flank, and the kidney was gently exteriorized using a cotton swab. Renal microcirculation was then observed and recorded using a two‐photon intravital microscope (Intravital Microscope, IVIM Technology).

### 2.13 mRNA Sequencing

Mouse kidney tissues (*N* = 4) were collected and immediately frozen in liquid nitrogen. Total RNA was extracted using Trizol reagent, and RNA concentration and integrity were assessed prior to library construction. After enrichment with oligo(dT)‐conjugated magnetic beads, mRNA was fragmented into 300–350 bp. Double‐stranded cDNA was prepared through sequential synthesis: first‐strand synthesis with random primers, followed by second‐strand synthesis. The cDNA was purified, end‐repaired, A‐tailed, and ligated to sequencing adapters. Fragments of 370–420 bp were size‐selected, PCR‐amplified, and purified to generate the final libraries. Library quality and effective concentration were assessed using a Qubit 2.0 Fluorometer, an Agilent 2100 Bioanalyzer, and qRT‐PCR, respectively. The edgeR package was used to identify differentially expressed genes. Significantly differentially expressed genes were those with |log_2_FC| > 1 and adj. *p < 0.05*. The biological functions and pathways associated with these genes were identified through KEGG and GO enrichment analyses. Novogene (Beijing, China) carried out the RNA sequencing.

### 2.14 Determination of Try and Kyn in Plasma and Kidney

Tissues were prepared as a 20% (w/v) tissue homogenate in physiological saline solution. Plasma and tissue homogenate samples (10 µL) were processed by adding 190 µL of acetonitrile containing the internal standard (propranolol at 0.2 µg/mL). The mixture was briefly vortexed and then centrifuged at 20 000g for 5 min. The supernatant was used for LC‐MS/MS analysis to determine concentrations of Kyn and Trp.

Chromatography was performed using a Zorbax C18 column (100 mm × 2.1 mm, 3.5 µm) at 37°C. Analytes were eluted with a gradient of acetonitrile and water containing 0.1% formic acid at a flow rate of 0.2 mL/min. Detection was carried out in the MRM positive ion mode, monitoring transitions of m/z 204.9 to 188 for Trp, m/z 208.8 to 192.1 for Kyn, and m/z 260.0 to 183.0 for the internal standard.

### 2.15 Immunofluorescence Assay for AhR Nuclear Translocation

Following fixation in 4% paraformaldehyde for 15 min at room temperature, cells were washed twice with PBS for 5 min each. Cells were then permeabilized with 100 µL of 0.3% Triton X‐100 for 15 min at room temperature. After permeabilization, cells were incubated with 5% BSA for 1 h at room temperature. Subsequently, cells were incubated with anti‐AhR mouse monoclonal antibody (1:50) overnight at 4°C. The next day, after re‐equilibration to room temperature, cells were stained with mouse fluorescent secondary antibody (1:500) for 45 min protected from light. After three washes with PBS, the nuclei were stained with Hoechst dye for 15 min. Cells were finally imaged using a laser scanning confocal microscope after thorough PBS washing.

### 2.16 Bacterial Clearance In Vitro

Two methods were used to determine the capability of bacterial clearance post‐treatment with IMM‐H018.

RAW 264.7 murine macrophages were plated in 24‐well plates at 5 × 10^5^ cells per well and divided into five groups: control, LPS, LPS + Epa (0.5 µM) + Siv (0.5 µM), LPS + IMM‐H018 (0.5 µM), and LPS + IMM‐H018 (2.5 µM) (*n* = 3). Cells were stimulated with LPS (1 µg/mL) for 24 h (except control).

The first method was the plate dilution (CFU) assay to assess macrophage phagocytic capacity. Cells were incubated with bacteria (MOI = 10) for 20 min to allow phagocytosis. Following PBS washes to clear extracellular bacteria, cells were lysed in 0.1% Triton X‐100 for 15 min. Cell lysates were subjected to stepwise 10‐fold dilutions in sterile PBS and then inoculated onto bacterial agar plates. Bacterial colonies were enumerated as colony‐forming units (CFUs) after overnight incubation at 37°C.

The second method was an immunofluorescence assay. For phagocytosis analysis, RAW 264.7 cells were plated in confocal dishes at 5 × 10^4^ cells per well. After pretreatment and LPS stimulation as described above, Escherichia coli (DH5α) transfected with pBS‐IdhGFP (Addgene, MA, USA) were added at MOI = 10 and incubated for 30 min. The cells were rinsed with PBS prior to fixation in 4% paraformaldehyde for 15 min, and labeled with Hoechst (1:10000) for nuclear visualization. Images were captured via a laser scanning confocal microscope (OLYMPUS FV 3000).

### 2.17 Cell Culture and Polarization Induction

The murine macrophage cell lines RAW264.7 and Ana‐1 purchased from the Cell Resource Center, Institute of Basic Medical Sciences, CAMS/PUMC were cultured under standard culture conditions (37°C, 5% CO_2_). Ana‐1 macrophages were maintained in RPMI‐1640, while RAW264.7 macrophages were grown in DMEM, with both media containing 10% FBS, 50 U/mL penicillin, and 50 U/mL streptomycin.

Aims to induce the M2 type, RAW264.7 and Ana‐1 macrophages were plated in 6‐well plates at 2 × 10^6^ cells per well. After adhesion, cells were divided into five groups: control, IL‐4, IL‐4 + Epa (0.5 µM) + Siv (0.5 µM), IL‐4 + IMM‐H018 (0.5 µM), and IL‐4 + IMM‐H018 (2.5 µM) (*n* = 3 per group). Cells were treated with the combination or IMM‐H018 and stimulated with IL‐4 (20 ng/mL) for 24 h to induce M2 polarization. Cells were harvested and subjected to flow cytometric analysis for the expression of CD86 and CD206. The following antibodies were obtained from BioLegend (San Jose, CA, USA): CD86‐PerCP/Cy5.5 (105026), and CD206‐APC (141708).

### 2.18 Western Blot

After collection and washing, kidney tissues or cells were lysed in chilled RIPA buffer (Solarbio, Beijing, China) with added protease and phosphatase inhibitors (Lablead, Beijing, China). Protein extracts (30 µg from whole‐cell lysates or 50 µg from tissue homogenates) were separated using SDS‐PAGE and then electroblotted onto PVDF membranes (IPVH00010, Merck). The membranes were blocked with 5% non‐fat milk for 1 h at room temperature and then incubated with primary antibodies at 4°C overnight. The next day, HRP‐linked secondary antibodies were applied to detect protein‐antibody interactions. Protein expression was measured using Meilunbio West Femto Maximum Sensitivity Substrate (MA0187‐3, Meilunbio). Antibodies employed in this study was following: anti‐IDO1(86630, CST), anti‐TF (44861, CST), anti‐AhR(MA1‐513, ThermoFisher), anti‐Histone 3(4499, CST), anti‐CYP1A1(13241‐1‐AP, Proteintech), β‐actin (TA‐09, SGB‐BIO), GAPDH (TA‐08, SGB‐BIO).

### 2.19 Pharmacokinetic Study

The plasma pharmacokinetics of IMM‐H018 were evaluated in male C57BL/6J mice (22–25 g) and Sprague‐Dawley (SD) rats (180–200 g). for oral administration (po), IMM‐H018 was suspended in 0.5% carboxymethylcellulose (CMC). For intravenous (iv) administration, IMM‐H018 was dissolved in 20% hydroxypropyl‐β‐cyclodextrin (HP‐β‐CD) with 10% DMSO. Mice and rats respectively, received IMM‐H018 by intragastric (ig) at 15 mg/kg or by iv at 1.5 and 0.5 mg/kg. Blood specimens were harvested at multiple designated time intervals (*n* = 3).

To assess tissue exposure of IMM‐H018 in an acute kidney injury (AKI) mouse model, male BALB/c mice (18‐20 g, *n* = 4) received IMM‐H018 by ig at 15 mg/kg/day for 3 successive days. Following the last administration, mice were challenged with LPS (10 mg/kg, intraperitoneal). 24 h after LPS administration, blood was collected and plasma was separated. Liver, kidney, and lung tissues were harvested and homogenized to prepare 25% (w/v) tissue homogenates. LC‐MS/MS was used to measure IMM‐H018 levels in plasma and tissue homogenates.

### 2.20 In Vivo Safety Evaluation

To preliminarily assess the in vivo safety profile of IMM‐H018, a 2‐week repeated‐dose toxicity study was conducted in both female and male SD rats by oral administration of IMM‐H018 at doses of 100 and 300 mg/kg/day. Body weight was monitored throughout the treatment period. At the end of the study, animals were sacrificed for hematological, serum biochemical, urinary, and coagulation analyses.

### 2.21 Statistical Analysis

Data are presented as the mean ± standard deviation (SD). Survival in the two subgroups was analyzed using the Kaplan‐Meier method, with group comparisons assessed by the log‑rank test. Nonparametric tests were used for data that did not follow a normal distribution. One‐way ANOVA was applied for multiple group comparisons, followed by the Bonferroni post hoc test. Significance was accepted at *p < 0.05*. All statistical analyses were performed using GraphPad Prism version 10.1.2 (La Jolla, CA, USA).

## 3 Results

### 3.1 Combination of Epa and Siv Prolongs the Survival of Experimental Sepsis Better than Treatment Alone

The mechanisms of IDO1 and NE involved in the immunosuppression, inflammation, and tissue damage caused by sepsis were briefly illustrated in Figure 1A,B. LPS‐induced sepsis and CLP models were used in the current study, respectively, and the detailed experimental procedure was presented in Figure 1C,D. For the LPS model, mice administered with Epa + Siv showed an improved 72h survival (42.7%, *p < 0.001*) compared with for pre‐treated with saline (6.7%), and Siv alone also significantly prolonged the survival (40%), but less than effective than combination (Figure 1E); Epa treatment slightly improved the survival of septic mice but no meaningful difference compared with saline (13.3%, *p < 0.05*). Similar results were found in CLP sepsis, but only co‐treatment had a significantly improved 72 h survival (50%) compared with saline treatment (6.7%). Mice treated by Siv alone had slightly prolonged survival (25%), but no significant difference was found (Figure 1F).

### 3.2 Combination of Epa and Siv Attenuates LPS‐Induced Inflammation and Immunosuppression in Mice Better Than Treatment Alone

Low dosage of LPS (10 mg/kg) can induce septic characteristics, manifested by cytokine storm and immunosuppression simultaneously. In the current study, the mice received i.p. injection of the 10 mg/kg LPS, and following treated by Epa, Siv and combination (the procedure was illustrated in Figure 2A). 24h later, the serum from each mouse was collected. As shown in Figure 2B, the production of major cytokines, including TNF‐α, MCP‐1, IL‐6 and IL‐10, was increased by LPS, and all three treatments decreased the serum cytokine concentrations, while the co‐treatment showed slightly better effect than both single treatment; especially for IL‐6, only co‐treatment significantly reduced its serum concentration, but not single treatment.

Immunosuppression was mostly characterized by significantly reduced peripheral lymphocytes cells, mainly due to lymphopenia caused by sepsis. As shown in Figure 2C, total blood cell count using Hematology Analyzer revealed that decreased lymphocyte number in blood was significantly increased by Epa and co‐treatment, but not Siv single treatment, and Epa + Siv had relatively better effect. However, three treatments did not change the cell number of peripheral monocytes and neutrophils (Figure 2C). By flow cytometry, same result was obtained that the significant decreased CD3^+^ cells in blood from LPS model group was also significantly restored by treatment with Epa and co‐treatment, but not Siv administration alone (Figure 2D). Another important immunosuppressive characteristic was thymus shrinkage and lymphocyte apoptosis in thymus, and by testing the relative thymus weight, which was shown in Figure 2D, we confirmed that the relative thymus weight was significantly reduced in the LPS model, and Epa single treatment and combination treatment increased the mean relative thymus weight by 18.3% and 33.8% respectively, although no statistical significance. By flow cytometry, we further demonstrated that LPS caused higher apoptosis of CD3^+^ lymphocytes in thymus, and although all three treatments reduced this trend, only combination treatment showed significant effect (*p < 0.01*, Figure 2E). The gating strategy was shown in Figure S1.

Upregulation of PD‐1 on T cells and monocytes were another hallmark for sepsis related immunosuppression [23]. Flow cytometric analysis revealed a significant increase in the proportion of PD‐1^+^ cells among total T cells (CD3^+^) and monocytes (CD11b^+^) in the spleen and blood of septic mice compared with controls (Figure 2G,H, Figure S3, the PD‐1 gating strategy was shown in Figure S2). The proportion of PD‐1^+^ cells among CD3^+^ T cells and CD11b^+^ monocytes was down‐regulated in all treatment groups, but co‐treatment had the best efficiency (Figure 2H, Figure S3). Besides the PD‐1^+^ percentage, flow cytometry also demonstrated that Epa and co‐treatment also could significantly reduce the mean fluorescence intensity (MFI) of PD‐1 expression on CD3^+^, CD4^+^ and CD8^+^ T lymphocytes in spleen, but not Siv treatment alone, and combination has best efficiency (Figure S5, with gating strategy in Figure S4).

### 3.3 Combination of Epa and Siv Attenuates LPS‐induced AKI More Effectively than Treatment Alone

AKI contributes to the lethality of sepsis, and we further investigated the kidney protection effect of Epa + Siv. First, the reduced body weights of mice induced by LPS were significantly reversed by co‐treatment, but not each single treatment (Figure 3A). Further the higher relative kidney weights were found in the model group, and was markedly decreased by co‐treatment (Figure 3B), but not single treatment neither (Figure 3B). By biochemical test, although three treatments all remarkably reduced BUN, serum β2‐MG and NGAL in septic mice, co‐treatment had best effect than each single treatment (Figure 3C). Pathological analysis by HE staining further confirmed that co‐treatment significantly preserved renal structural integrity by reducing tubular epithelial edema (green arrows), interstitial inflammatory infiltration (blue arrows), and microthrombosis (red arrows) in septic mice (Figure 3D), which was also manifested by tubular injury score analysis (Figure 3E). Immunohistochemistry using NGAL also demonstrated that widely distributed NGAL staining in interstitial areas was significantly reduced by co‐treatment, which is better than single alone too (Figure 3D,F). All these results suggested that co‐treatment had most beneficial effect on septic AKI. To further investigate the potential effect and underlying mechanisms of Epa + Siv on AKI, we perform the RNA‐seq analysis using renal cortex. The coagulation and consequent ischemia and hypoxia contribute to the renal dysfunction, manifested by reduced oxidation and increased glycolysis (Figure S6). By KEGG analysis, the top 10 down‐regulated KEGG pathways by Epa + Siv was shown in Figure 3G, and SA‐AKI related pathways, such as complement and coagulation pathway, Leukocyte transendothelial migration, apoptosis, tryptophan metabolism pathways were significantly inhibited. Reduced glycolysis and HIF‐1 signaling pathway were also confirmed, and proved that low oxygen supply caused by low perfusion was improved. GSEA analysis also demonstrated that Epa + Siv upregulated the oxidative phosphorylation in the renal cortex compared with model group, which was consistent with reduced glycolysis (Figure 3H). On the other side, KEGG (Figure 3G) and GSEA analysis (Figure 3I) both demonstrated that Epa + Siv reduced the ER stress which also was one of important contributor to the septic AKI pathophysiology.

**FIGURE 3:**
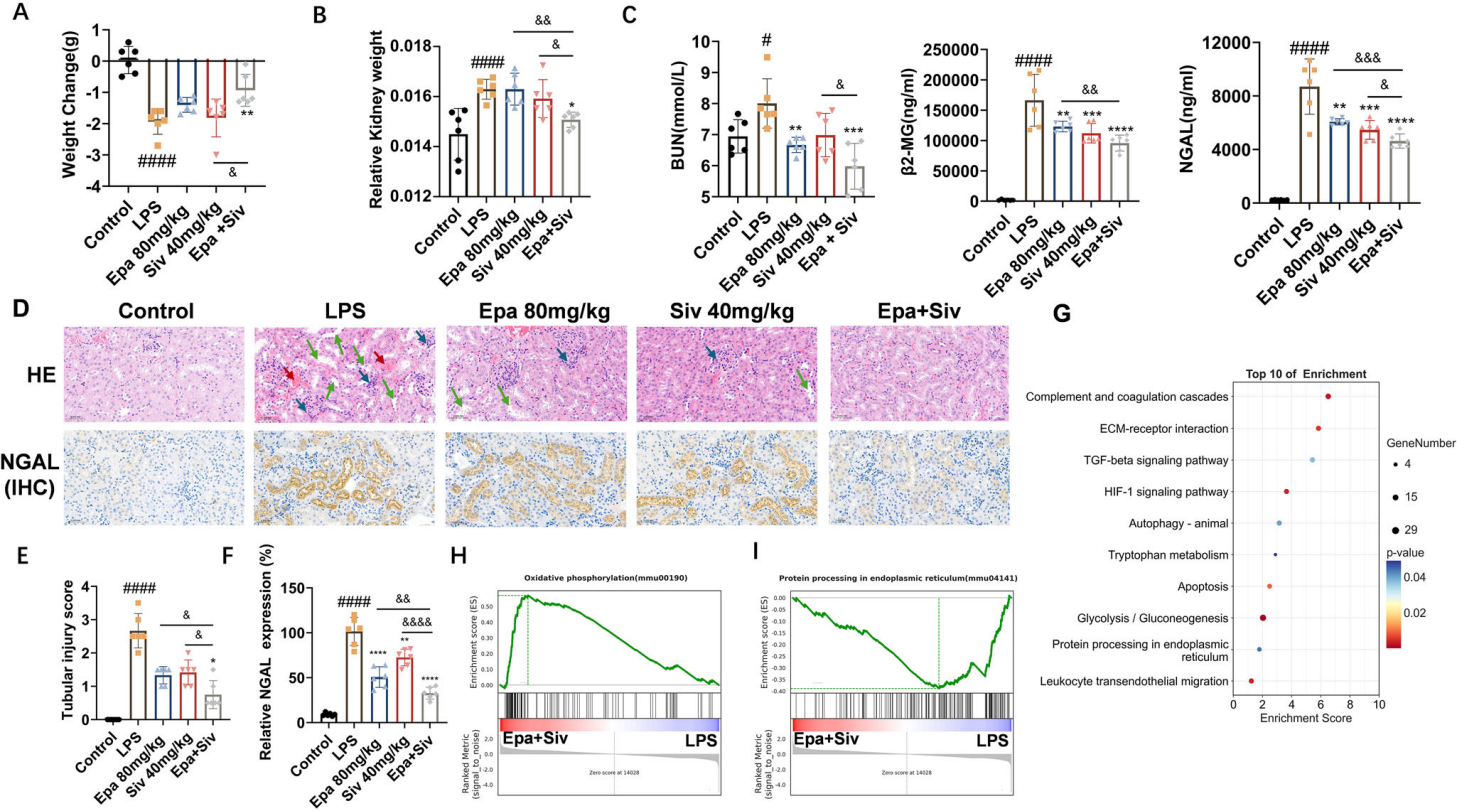
Combination of Epa and Siv attenuates LPS‐induced AKI better than treatment alone. (A) Co‐treatment significantly reversed the body weight loss induced by LPS. B. Co‐treatment significantly reversed the LPS‐induced elevation of relative kidney weight. (C) Serum BUN, β2‐MG, and NGAL levels were markedly decreased by co‐treatment. (D) H&E staining revealed reduced tubular injury (green arrows), inflammatory infiltration (blue arrows), and microthrombosis (red arrows) after co‐treatment. (E) NGAL immunostaining in renal tissue was also significantly decreased following co‐treatment. Data are presented as mean ± SD. *p < 0.05, **p < 0.01, ***p < 0.001, ****p < 0.0001 vs. LPS group. *^#^p < 0.05, ^##^p < 0.01, ^####^p < 0.0001* vs. Control group. *^&^p < 0.05*, *^&&^p < 0.01, ^&&&^p < 0.001* vs Epa + Siv group.

### 3.4 Development of a Novel Specific Dual IDO1/NE Inhibitor IMM‐H018 In Vitro

In order to evaluate our proposed therapy strategy for sepsis and facilitate the future clinical application, IDO1 and NE dual inhibitor was developed. The design combined the strategies of fragment‐based drug design and substrate mimicking. First, IDO1 catalyzed the oxidation of tryptophan to N‐formylkynurenine, which contains a core structure of 2‐aminobenzoyl (bold blue line, Figure 4A). Interestingly, the core structure of 2‐aminobenzoyl was also presented in the structure of a known NE inhibitor Sivelestat (Figure 4B). This observation indicated that the fragment of 2‐aminobenzoyl can be used for the design of IDO1/NE dual inhibitor. Next, in our previous study we reported 2‐benzylsulfinyl‐benzoxazoles as potent and selective IDO1 inhibitors (Figure 4C) [39, 40]. However, 2‐benzylsulfinyl‐benzoxazoles does not show any inhibitory activity against NE with an IC_50_ over than 50µM. In order to obtain the NE inhibitory activity, the benzoxazole ring was broken to mimic the core structure of 2‐aminobenzoyl. The optimization eventually rendered the discovery of a series of 2‐(thiocarboxyamino) benzoic acids with enhanced IDO1 inhibitory activity while gaining inhibitory activity against NE. The chemical structures of the IPK‐series compounds, which represent a series of in‐house synthesized derivatives derived from the patented 2‐benzylsulfinyl‐benzoxazole scaffold, are provided in Supplementary Table S1. 2‐((((4‐chlorobenzyl) thio) carbonyl) amino) benzoic acid, designated as IPK‐006 and named IMM‐H018, was used in this study as IDO1/NE dual inhibitor (Figure 4C). By cell‐based enzyme activity screening assay, the IC_50_ of IMM‐H018 was for 41.4nM and 0.82µM for IDO1 and NE respectively (Figure 4C, Table 1). In addition, both IDO1 and TDO catalyze the oxidation of L‐tryptophan to N‐formyl kynurenine, we also examined the cross reactivity of IMM‐H018 against IDO1 and TDO. IMM‐H018 shows weak inhibitory activity against TDO with the IC_50_ over than 10µM. Therefore, IMM‐H018 exhibits good selectivity against IDO1 and TDO.

**FIGURE 4:**
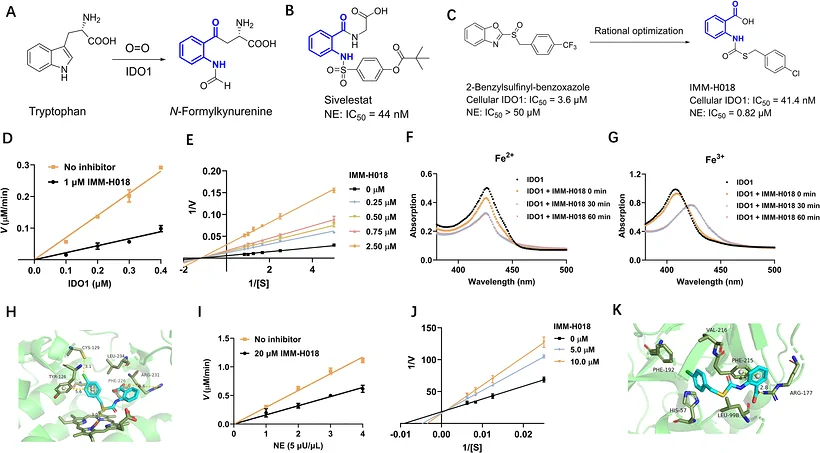
Discovery and characterization of IMM‐H018 as a novel dual IDO1/NE inhibitor in vitro. (A) The core 2‐aminobenzoyl structure (blue) in the IDO1‐catalyzed product N‐formylkynurenine. (B) Presence of the 2‐aminobenzoyl core in the known NE inhibitor sivelestat. (C) Chemical structures of the parent IDO1 inhibitor (2‐benzylsulfinyl‐benzoxazole) and the optimized dual inhibitor IMM‐H018 (2‐((((4‐chlorobenzyl) thio) carbonyl) amino) benzoic acid). Cell‐based IC_50_ curves of IMM‐H018 against IDO1 (41.4 nM) and NE (0.82 µM). D‐E. Enzyme kinetic analyses of IMM‐H018 against IDO1: (D) Reversible inhibition; (E) Non‐competitive inhibition with respect to D‐tryptophan. (F, G) UV–vis spectroscopy showing that IMM‐H018 binds to the ferric (Fe^3^^+^) heme of IDO1, clasifying it as a type III inhibitor. (H) Docking model of IMM‐H018 in IDO1 (PDB: 6E40), illustrating interactions with heme iron, Tyr126, and Arg231. (I, J) Enzyme kinetic analyses of IMM‐H018 against NE: (I) Reversible inhibition; (J) Competitive inhibition with respect to the peptide substrate. (K) Docking model of IMM‐H018 in NE (PDB: 1B0F), showing salt bridge formation with Arg177 and hydrophobic interactions in the active site.

**TABLE 1:** Results of enzyme activity screening.

Compound number	IDO1 IC_50_ (µM)	TDO IC_50_ (µM)	NE IC_50_ (µM)
IPK‐003	0.2	>100	0.56
**IMM‐H018(IPK‐006)**	**0.0414**	**10.3**	**0.82**
IPK‐009	0.889	18.3	1.67
NLG919	0.064	0.085	25.9
**Epacadostat**	**0.023**	**26.4**	**>50**
**Sivelestat**	**>50**	**>50**	**0.079**

The inhibition modes of IMM‐H018 toward the targets of IDO1 and NE was investigated. First, we studied the interaction between IMM‐H018 and IDO1. IDO1 enzyme kinetic studies showed that the fitted lines of no inhibitor and IMM‐H018 were crossed in the plot, which means IMM‐H018 is a reversible inhibitor of IDO1 (Figure 4D). Further enzyme kinetic study also revealed that IMM‐H018 is a non‐competitive inhibitor of the substrate of D‐tryptophan since the fitted lines of various concentrations of IMM‐H018 groups were intersected in the x‐axis of the plot (Figure 4E). IDO1 is a heme containing enzyme, and the cofactor of heme is located in the active center of IDO1. Heme UV absorption spectra are often used to exhibit the direct binding between the inhibitor and the iron ion in the active center of IDO1 [41]. We carried out the classic heme binding study to explore whether IMM‐H018 interacted with ferrous or ferric forms of IDO1 (Figure 4F). UV–vis spectroscopy studies showed that the addition of IMM‐H018 into the IDO1 solution changes the Soret band of heme for the ferric form of IDO1 but not ferrous form of IDO1. Notably, the substrate free ferric enzyme needs to turn over to the active ferrous state in order to function the catalysis. This demonstrated the IMM‐H018 interacts with Fe3^+^‐heme to inhibit the catalytic function of IDO1 (Figure 4G), and this also defined IMM‐H018 as type III IDO1 inhibitor. Moreover, IMM‐H018 was docked into the crystal structure of IDO1 (PDB code:6E40) using BIOVIA Discovery Studio 2023. The predicted docking mode showed in Figure 4D, which revealed that the sulfur atom of IMM‐H018 bound with the heme iron. The 4‐chlorobenzyl moiety of IMM‐H018 occupied pocket A and formed an edge to face π‐π stacking interaction with Tyr126 and hydrophobic interactions with other amino residues in pocket A. The benzoic acid scaffold of IMM‐H018 stretched to pocket B and formed hydrogen bond with Arg231.

Next, we investigated the interaction between IMM‐H018 and NE. We carried out the enzyme kinetic study to show that IMM‐H018 is a reversible inhibitor of NE as the fitted lines of no inhibitor and IMM‐H018 were intersected in the plot (Figure 4I). In addition, our studies also showed that IMM‐H018 was a competitive inhibitor of the peptide substrate of NE. The Lineweaver‐Burk plot showed that the fitted lines of various concentrations of IMM‐H018 groups were intersected in the y‐axis of the plot (Figure 4J). In addition, IMM‐H018 was docked into the crystal structure of NE (PDB code: 1B0F) using BIOVIA Discovery Studio 2023 (Figure 4K). The benzoic acid scaffold of IMM‐H018 formed a salt bridge with Arg177 and a π‐π stacking interaction with Phe215. The 4‐chlorobenzyl moiety occupied the hydrophobic pocket composed by Val216, Phe192, His57 and Leu 99B.

To summarized, IMM‐H018 is a dual inhibitor of IDO1/NE. IMM‐H018 acts as reversible inhibitor toward both IDO1 and NE, and there is no covalent bond formed between IMM‐H018 and the enzymes of IDO1 and NE. IMM‐H018 is non‐competitive inhibitor to the substrate of D‐tryptophan of IDO1, and it is competitive inhibitor of the peptide substrate of NE.

### 3.5 IMM‐H018 Reduces the Cytokine Storm and Restores the Immune Homeostasis in LPS Induced Septic Mice via Inhibiting NE and IDO1 Activity In Vivo

Same LPS (10mg/kg) induced unlethal sepsis model was applied to determine the beneficial effect of IMM‐H018 on cytokine storm and immunosuppression, and three dosages (15, 30, and 60mg/kg) of IMM‐H018 were administered, as well as Epa (80mg/kg) and Siv (40mg/kg) co‐treatment as positive control. The detailed procedure was illustrated in Figure 5A. First, the IDO1 and NE activity in circulation was confirmed significantly up‐regulated in the current septic mice. The Kyn/Tyr ratio from the serum of each animal was calculated to analyze the efficiency of IMM‐H018 on IDO1 activity in vivo. Results demonstrated that IMM‐H018 can significantly decrease the Kyn/Tyr ratio dose dependently in vivo, and at 60mg/kg dosage had no difference with EPA at 80mg/kg (Figure 5B). Meanwhile, by neutrophil elastase activity assay (ab204730, abcam), we also confirmed that IMM‐H018 inhibited peripheral NE activity in a dose‐dependent manner, although its effect was slightly lower than Siv (Figure 5C). All these results showed that IMM‐H018 shared dual inhibitory effects on both enzyme activity of IDO1 and NE in vivo.

**FIGURE 5:**
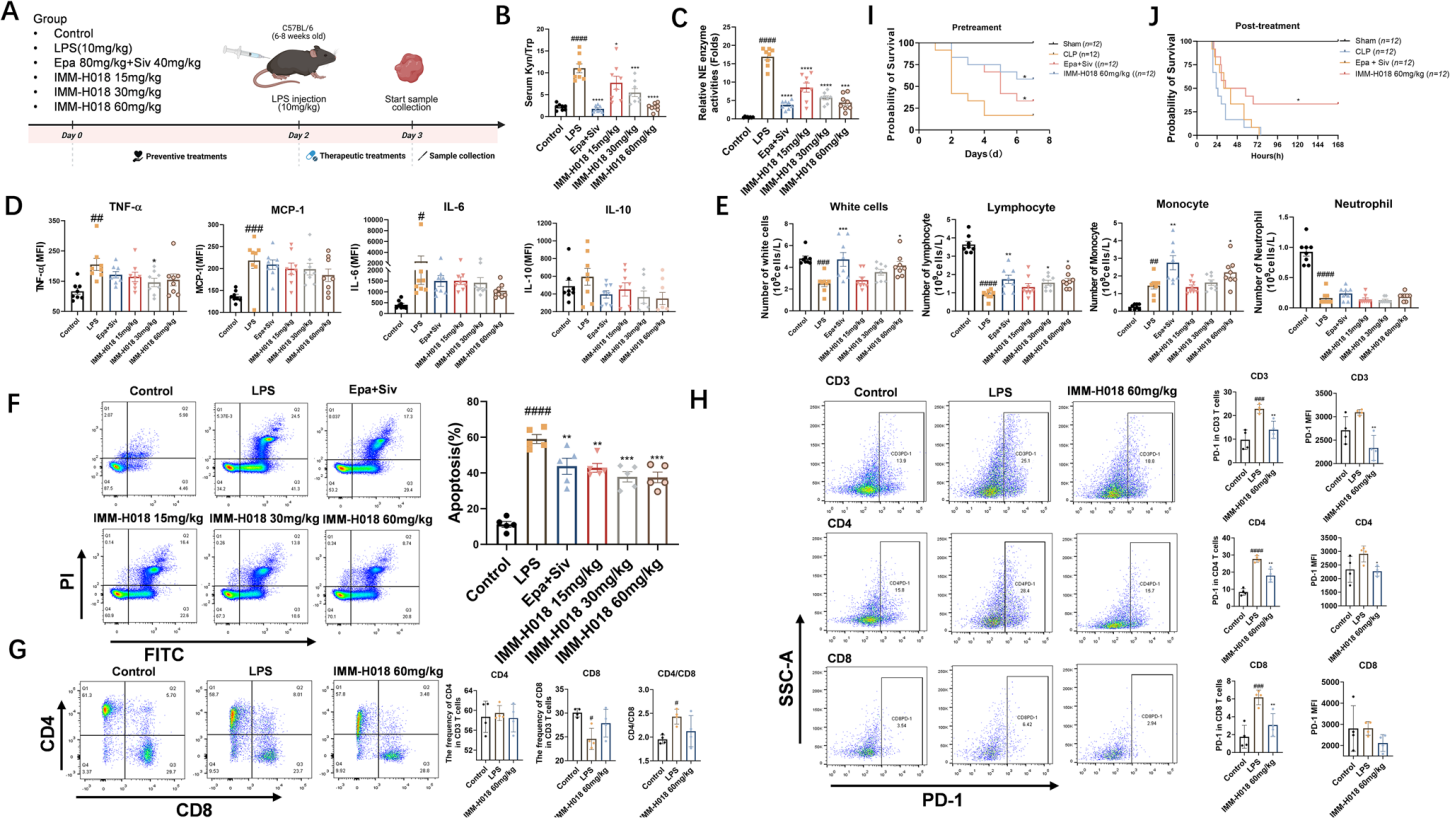
IMM‐H018 ameliorates LPS‐induced sepsis and improves survival by inhibiting NE and IDO1 in vivo. (A) Experimental timeline for LPS‐induced non‐lethal sepsis and drug administration. (B) Serum Kyn/Trp ratio reflecting IDO1 activity in mice. (C) Neutrophil elastase (NE) activity in peripheral blood. (D) Serum concentrations of TNF‐α, MCP‐1, IL‐6 and IL‐10. (E) Peripheral white cells, lymphocyte, monocyte and Neutrophil counts. (F) Apoptosis of thymic CD3^+^ T cells. (G) Peripheral blood CD4/CD8 T cell ratio. (H) PD‐1 expression on CD3^+^, CD4^+^, and CD8^+^ T cells. I. Survival curve of CLP‐induced lethal sepsis after IMM‐H018 post‐treatment. (J) Survival curve of CLP‐induced lethal sepsis after IMM‐H018 treatment initiated 4 h after CLP surgery. Data are presented as mean ± SD. *p < 0.05, **p < 0.01, ***p < 0.001, ****p < 0.0001 vs. LPS or CLP group. *^#^p < 0.05, ^###^p < 0.001, ^####^p < 0.0001* vs. Control group.

Cytokine assay demonstrated that IMM‐H018 treatment significantly ameliorated cytokine production dose dependently, including TNF‐α, MCP‐1, IL‐6 and IL‐10, which was effective than combination (Figure 5D). By complete blood counting, IMM‐H018 significantly increased the total white cell number, mostly due to the increased lymphocytes and monocytes (Figure 5E), and its restoring immune homeostasis at 60mg/kg was almost same as co‐treatment. By flow cytometry, IMM‐H018 significantly reduced the apoptosis of thymus T cells, and slightly better than Epa + Siv at 60mg/kg (Figure 5F). Further, IMM‐H018 at 60mg/kg significantly decreased peripheral CD4/CD8 ratio (Figure 5G) and reduced the PD‐1 expression percent and MFI on CD3^+^, CD4^+^ and CD8^+^ T cells (Figure 5H). All these suggested that IMM‐H018 not only could effectively reduce the cytokine storm, but also reversed the immune suppression, and slightly better than Epa + Siv.

Further, the effect of pre‐treatment of IMM‐H018 (60mg/kg) on survival of lethal sepsis were tested by CLP mice under same procedure as Figure 1D, and Epa + Siv was used as positive control. Mice pretreated with IMM‐H018 showed a significantly improved survival rate (58.3%, *p < 0.05*) compared with vehicle treatment group (8.3%), showed better trend than co‐treatment (33.3%, *p < 0.05*) (Figure 5I). In contrast, in the therapeutic treatment setting (Figure 5J), post‐CLP administration of IMM‐H018 resulted in a survival rate of 33.3%(*p < 0.05*), whereas all mice in both the vehicle and combination treatment groups succumbed during the observation period.

### 3.6 IMM‐H018 Alleviates Multi‐Organ Injury and Protects Against Sepsis‐Associated Acute Kidney Injury

As sepsis is a systemic syndrome involving multiple organs, we additionally evaluated the effects of IMM‐H018 on multi‐organ injury and systemic safety. As shown in Figure S7A, septic mice exhibited increased kidney, lung, and liver indexes, whereas IMM‐H018 treatment ameliorated these organ index alterations to varying degrees, with the most pronounced improvement observed in the kidney. In addition, serum ALT and AST levels were not further elevated after IMM‐H018 treatment (Figure S7B). Moreover, bronchoalveolar lavage fluid (BALF) protein concentration and peripheral NEUT% were markedly increased in septic mice, while IMM‐H018 treatment significantly reduced these inflammatory indicators (Figure S7C), indicating that IMM‐H018 also exerted protective effects against systemic inflammation and lung injury during sepsis. Consistent results were obtained in the therapeutic treatment model, in which IMM‐H018 also alleviated systemic inflammation and multi‐organ injury (Figure S8).

Among sepsis‐associated organ injuries, sepsis‐associated acute kidney injury (SA‐AKI) is one of the most common and life‐threatening complications, and renal microcirculatory dysfunction and microthrombosis are recognized as major pathological drivers of SA‐AKI. In addition, previous clinical studies demonstrated that elevated circulating IDO1 activity is closely associated with declined renal function in septic patients [42, 43]. Therefore, the kidney was selected as the primary target organ for mechanistic investigation of IMM‐H018.

The renal function of IMM‐H018 was investigated using the same batch of LPS (10mg/kg) induced septic mice. By biochemical examination, IMM‐H018 significantly decreased the peripheral serum concentration of BUN, β2‐MG, and NGAL dose dependently, and at the 60mg/kg dosage, its effect was better than Epa + Siv (Figure 6A). Based on previous clinical publications in which circulating IDO1 activity was associated with declined renal function in septic patients [44, 45], we performed the correlation study using the septic mice. As shown in Figure S9, the Kyn/Tyr concentration was significantly positively correlated with BUN, which further confirmed same consistency in septic mice. We then performed HE staining to assess renal structural damage in septic mice. As shown in Figure 6B, septic injury induced severe renal tissue injury, which was similar as mentioned in the Figure 3D; however, IMM‐H018 treatment dose‐dependently ameliorated these morphological alterations (Figure 6B,C). Immunohistochemistry using NGAL also demonstrated that IMM‐H018 significantly ameliorated the kidney impairment dose dependently (Figure 6B,D). Kidney perfusion was compared between vehicle control and IMM‐H018 treatment groups, given the role of renal hypoperfusion as a key driver of septic renal dysfunction. MSOT‐based detection of intrinsic NIR‐absorbing hemoglobin is widely recognized as a reliable technique for quantifying organ perfusion. As shown in Figure 6E,F, total hemoglobin intensity in the kidneys was markedly decreased after LPS challenge relative to sham group. This reduction was markedly reversed by IMM‐H018 treatment, suggesting better‐preserved renal perfusion was recovered, and effect of IMM‐H018 was better than co‐treatment of Epa and Siv.

**FIGURE 6:**
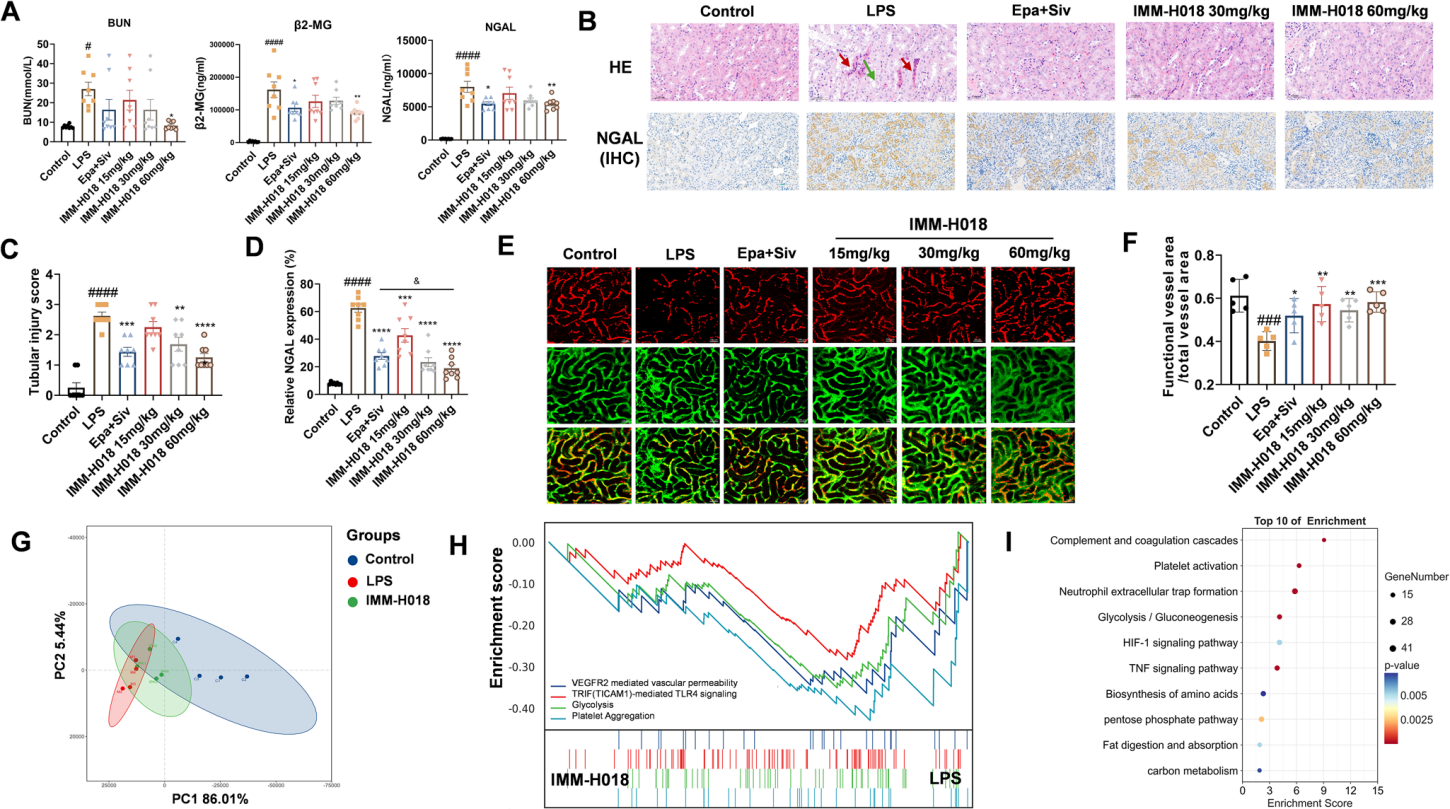
IMM‐H018 improves renal function and restores renal perfusion in LPS‐induced septic AKI. (A) Dose‐dependent reduction of serum BUN, β2‐MG, and NGAL by IMM‐H018 in septic mice, with 60 mg/kg IMM‐H018 outperforming Epa + Siv combination. (B–D) HE staining, NGAL immunohistochemistry and semi‐quantification showing IMM‐H018 dose‐dependently alleviates LPS‐induced renal pathological damage, and tubular injury is indicated with green arrows, and microthrombosis is indicated with red arrows (E, F) MSOT detection and quantification of renal total hemoglobin intensity, indicating IMM‐H018 improves renal perfusion in septic mice, superior to Epa + Siv treatment. (G) PCA analysis of renal cortex mRNA sequencing profiles. (H) GSEA enrichment analysis of IMM‐H018‐regulated differential genes. I. KEGG enrichment analysis of the top down‐regulated pathways by IMM‐H018. Data are presented as mean ± SD. *p < 0.05, **p < 0.01, ***p < 0.001, ****p < 0.0001 vs. LPS group; *^#^p < 0.05, ^###^p < 0.001, ^####^p < 0.0001* vs. Control group. *^&^p < 0.05* vs. Epa + Siv group.

mRNA sequencing using kidney cortex tissue was performed to further verify the underlying mechanisms of IMM‐H018's renal protection, and the PCA analysis demonstrated that IMM‐H018 treatment group significantly distinguished from the model group, and adjacent to the normal control group (Figure 6G). The top 100 differential up or down regulated mRNA (model vs control) reversed by IMM‐H018 was shown in Figure S10. Based on these reversed differential genes, GSEA analysis showed that IMM‐H018 treatment markedly downregulated TLR‐4 mediated inflammation process, glycolysis, platelet aggregation and vascular permeability, which were major pathological risk factors for SA‐AKI (Figure 6H). The KEGG results further revealed that top 10 enriched down‐regulated pathways by IMM‐H018 compared with model groups containing major platelet activation, HIF signaling pathway, NETs formation, TNF signaling pathway, and complement and coagulation cascades (Figure 6I), which are also major pathological risk factors. Altogether, these data indicated that IMM‐H018 represents a promising therapeutic agent for selectively alleviating SA‐AKI through anti‐inflammation and microthrombosis, and enhancing blood perfusion in kidneys.

### 3.7 IMM‐H018 Reduces the Renal Microthrombosis Through Inhibiting Tissue Factor Production via IDO1‐Kyn‐AhR‐TF Axis

Experimental data and mRNA sequencing showed that IMM‐H018 improved blood perfusion and reduced the coagulation in the kidneys, and microthrombosis contributed to this pathological condition. Further, by immunohistochemistry, we demonstrated that IMM‐H018 remarkably reduced the deposition of Fibrinogen in kidneys, mostly in glomeruli (Figure 7A), and further confirmed that IMM‐H018 inhibited renal microthrombosis. NETs also could cause microthrombosis in vessels in kidneys; therefore, by IHC, we also demonstrated that IMM‐H018 could significantly reduce the CitH3 formation in the glomeruli dose dependently (Figure 7A).

**FIGURE 7:**
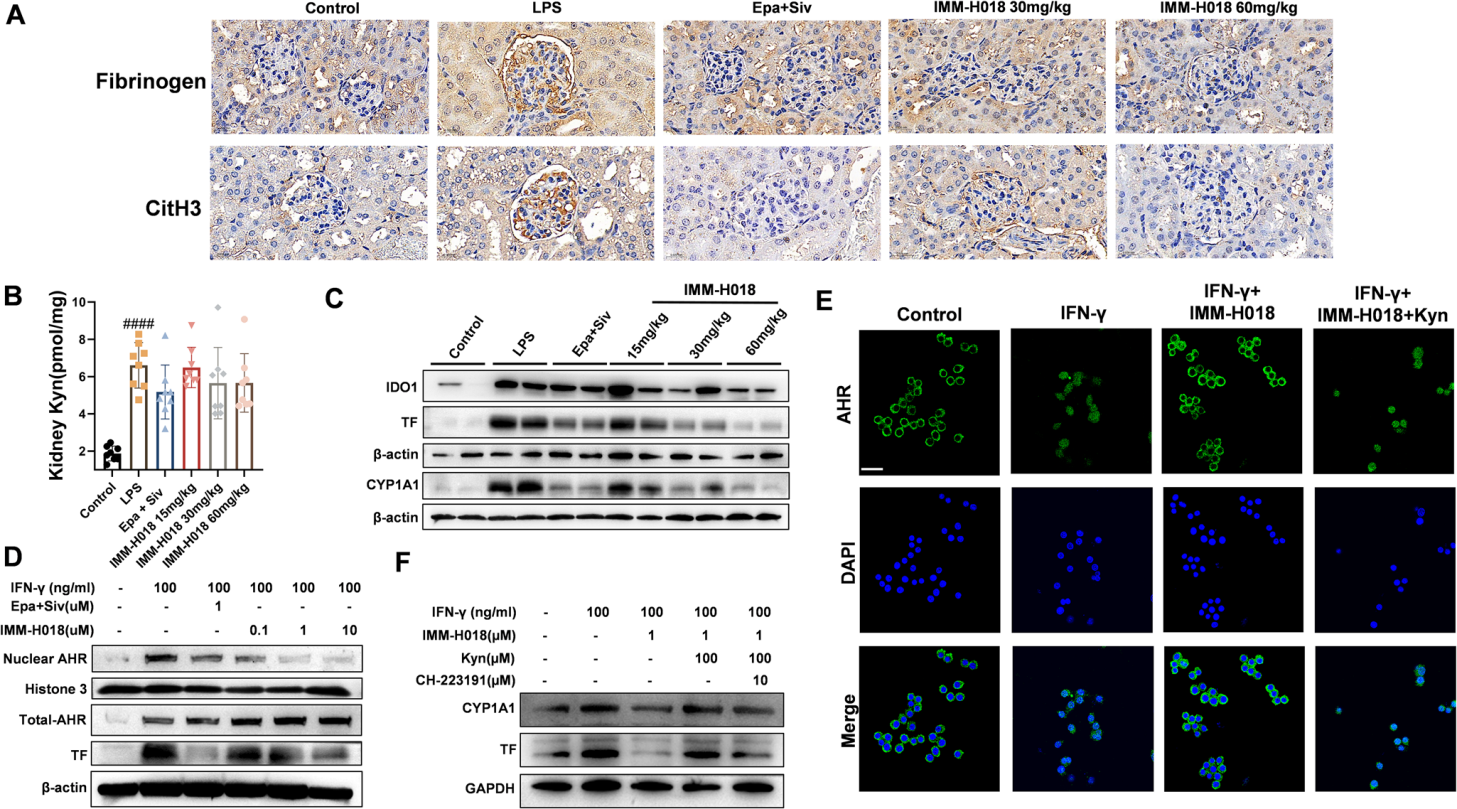
IMM‐H018 reduces renal microthrombosis via inhibiting NETs formation and IDO1‐Kyn‐AhR‐TF axis. (A) Immunohistochemical staining of fibrinogen deposition and CitH3, a marker of NETs formation, in renal tissues. (B) Renal kynurenine (Kyn) levels in mice. (C) Western blot analysis showing IDO1, tissue factor (TF), and CYP1A1 levels in renal cortex tissues. (D) In vitro analysis of AhR nuclear translocation and TF production in macrophages under IFN‐γ stimulation with IMM‐H018 treatment. (E) Immunofluorescence images showing AhR nuclear translocation (co‐localization with DAPI) and rescue by exogenous Kyn addition. Scale bars, 20 µm. (F) Western blot analysis of CYP1A1 and TF expression in macrophages under different treatments. Data are presented as mean ± SD. *^####^p < 0.0001* vs. Control group.

To evaluate the activation of the IDO1‐Kyn pathway in septic kidneys, renal Kyn levels were measured. As shown in Figure 7B, Kyn concentrations in the kidneys were significantly increased in septic mice, indicating activation of the IDO1‐Kyn pathway. Nevertheless, the effect was less pronounced in renal tissue compared with peripheral blood. IMM‐H018 treatment induced a mild reduction in renal Kyn content (approximately 15.0%) at doses of 30 and 60 mg/kg, without statistical significance. Epa decreased renal Kyn levels by 21.7%, and this change also failed to show statistical significance.

TF is initiator of microthrombosis, and IDO1‐Kyn‐AhR axis activation contributes to the TF over‐production in kidneys during sepsis [29]. Western blot using renal cortical tissue homogenate demonstrated that, the expression levels of IDO1, TF, and CYP1A1, a classical downstream target gene of AhR activation, were significantly increased in septic mice. Treatment with IMM‐H018 markedly reduced the expression of all three proteins, showing greater efficacy than Epa + Siv (Figure 7C). Macrophages are one of major cells which secret the TF. As shown in Figure 7D, IFN‐γ stimulation markedly promoted AhR nuclear translocation and increased TF expression in macrophages, indicating activation of AhR signaling. By IMM‐H018 treatment, the translocation of AhR was significantly reduced, and TF production was down‐regulated dose‐dependently. Further by immunofluorescence, we also proved that IMM‐H018 significantly inhibited the AhR translocating into the nucleus, while Kyn addition remarkably abolished such inhibitory effect as rescue assay (Figure 7E). To further confirm the role of AhR in regulating TF expression, Raw 264.7 cells were treated with the selective AhR antagonist CH223191. As shown in Figure 7F, CH223191 significantly attenuated Kyn‐induced upregulation of both CYP1A1 and TF, confirming that AhR activation is necessary for TF overproduction. Collectively, these results demonstrate that AhR mediates TF expression downstream of IDO1‐Kyn signaling, and that IMM‐H018 reduces TF overproduction by targeting IDO1 to block AhR activation, establishing the IDO1‐Kyn‐AhR‐TF axis as a key pathway in sepsis‐induced renal microthrombosis.

### 3.8 IMM‐H018 Significantly Improves Bacterial Clearance via Enhances Phagocytosis and Antigen Presentation of Macrophages in Murine Two‐Hit Sepsis

A key advantage of IMM‐H018 in the treatment of sepsis lies not only in its ability to inhibit sepsis‐induced inflammation but also in its potential to restore immune homeostasis, thereby helping to prevent secondary infections‐a critical concern for improving sepsis prognosis. Aims to confirm this advantage, a modified simple two‐hit sepsis model was established to mimic the second infection, and investigate whether IMM‐H018 could boost the immunity to clean the bacterium invasion. By preliminary study, as shown in Figure S11, the immunosuppression status in mice were still maintained after 72h later of LPS (10mg/kg) injection, therefore, at this time point, the E.coli was intraperitoneal injected, and the detailed procedure was shown in Figure 8A. 24h later, using bacterial plate cloning, the mice received IMM‐H018 exhibited a significantly reduced bacterial burden in peripheral blood relative to CMC‐Na‐treated controls, even at 15mg/kg dosage (*p < 0.01*) (Figure 8B).

**FIGURE 8:**
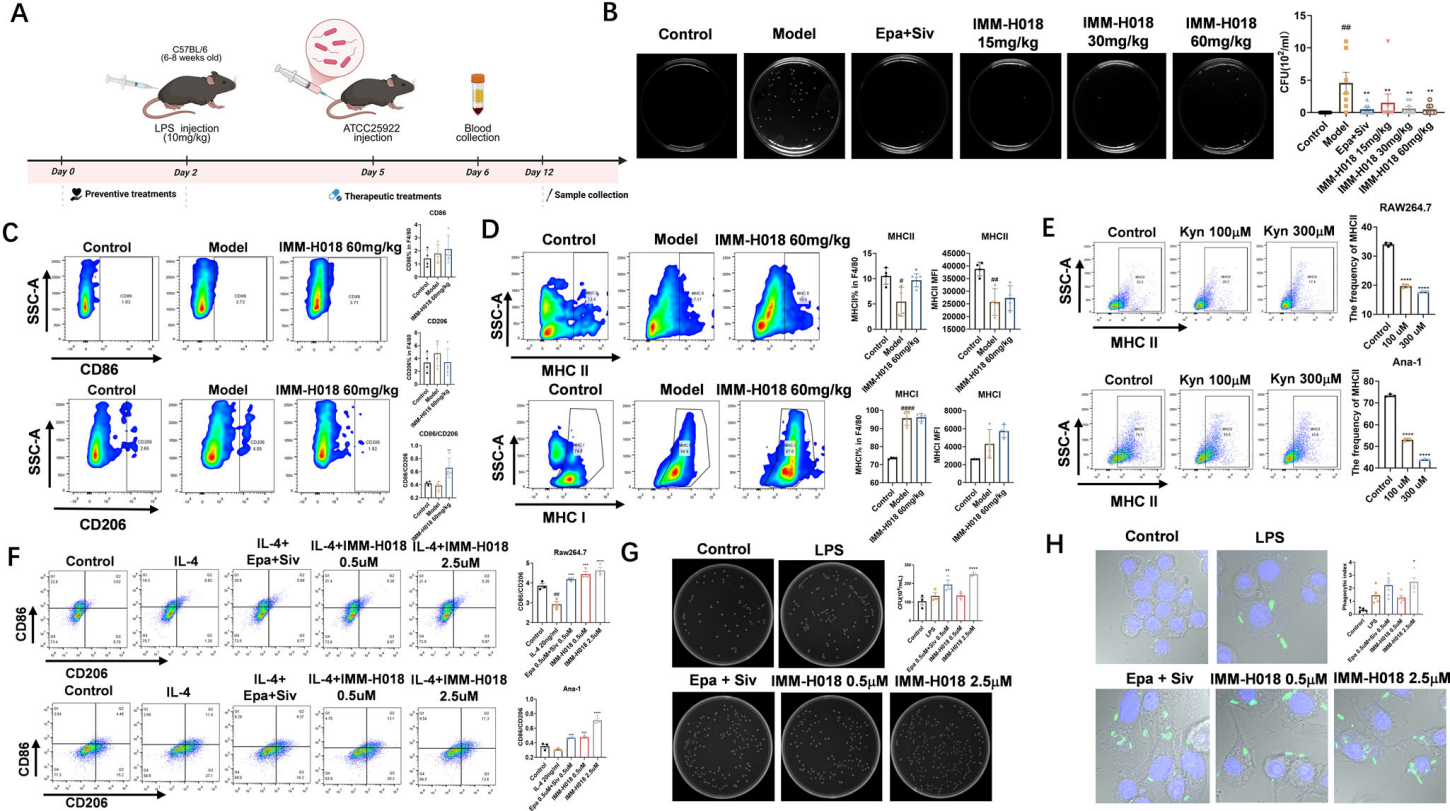
IMM‐H018 enhances bacterial clearance and restores macrophage function in two‑hit septic mice. (A) Schematic of the two‑hit sepsis model and experimental procedure. (B) Bacterial burden in peripheral blood after *E. coli* challenge. (C) Flow cytometric analysis of CD86^+^ and CD206^+^ macrophages in peripheral blood. (D) Expression of MHC II on peripheral macrophages. (E) Effects of kynurenine (Kyn) on CD206 and MHC II levels in RAW264.7 and Ana‐1 cells. (F) IMM‐H018 reverses IL‑4‑induced M2 polarization and MHC II downregulation in macrophages. (G, H) Phagocytosis and bactericidal activity of RAW264.7 cells pretreated with IMM‐H018. Data are presented as mean ± SD. *p < 0.05, **p < 0.01 vs. Model/Control group. *^#^p < 0.05, ^##^p < 0.01, ^###^p < 0.001, ^####^p < 0.0001* vs. Control group.

Macrophages played the key role to clear the bacterium burden, most through direct phagocytosis and antigen presentation. We isolated monocyte/macrophages from four mice each group, and the M1 and M2 biomarker, CD86 and CD206 were determined by flow cytometry, and gating strategy was provided in supplemental documents (Figure S12A). We could find that IMM‐H018 significantly reduced CD206^+^ ratio, and slightly increased CD86^+^ cells ratio (Figure 8C). In addition to phagocytosis, antigen presentation is another key function that enables macrophages to aid in bacterial clearance, and we further evaluate how the IMM‐H018 regulated the antigen presentation effect of macrophages. The downregulation of MHCII is a crucial indicator of sepsis‐induced immunosuppression, which impairs the function of the adaptive immune system, and this decreased trend was reversed by IMM‐H018 treatment (Figure 8D). However, The MHCI ratio in peripheral macrophages was not remarkably changed by IMM‐H018 treatment (Figure 8D), and gating strategy was provided in Figure S12B. By in vitro study, Kyn addition dose dependently decreased MHCII expression of RAW274.7 and Ana‐1, further proved that Kyn accumulation was one of mechanisms for macrophage immunosuppression (Figure 8E). By flow cytometry, the increased M2 ratio induced by IL‐4 in RAW264.7 and Ana‐1 was markedly reversed via IMM‐H018 treatment dose‐dependently (Figure 8F). We also confirm that the RAW264.7 treated by IMM‐H018 could eliminate the bacterium effectively, through plating method (Figure 8G) and direct observation using fluorescence labelling bacteria under microscopic (Figure 8H).

### 3.9 IMM‐H018 Ameliorates Inflammation, Immunosuppression and AKI in the Two‐Hit Sepsis and Prevents AKI and AKI‐CKD Transition

Two‐hit experimental sepsis caused more serious sepsis characteristics and organ injuries, which were demonstrated by higher production of inflammatory cytokines, lower lymphocyte count, and higher relative weights. By IMM‐H018 treatment, the inflammation was significantly reduced (Figure 9A), the reduced lymphocytes were recovered, and its effect was better than co‐treatment (Figure 9B). Meanwhile, IMM‐H018 treatment significantly reduced the lung, liver, kidney relative weight, and increased the thymus relative weight (Figure 9C). By HE staining and IHC, we confirmed that IMM‐H018 reduced the tubular injury score in two‐hit sepsis and NGAL expression in Tubulointerstitium (Figure 9D,E). More importantly, the AKI to CKD is an important clinical concern, and Kyn accumulation was considered to induce the fibrosis in the kidney. Immunohistochemistry using fibronectin demonstrated that IMM‐H018 treatment significantly reduced the fibronectin deposition in tubular arear, therefore suggested that IMM‐H018 treatment could possibly slow down AKI to CKD transition during sepsis (Figure 9F).

**FIGURE 9:**
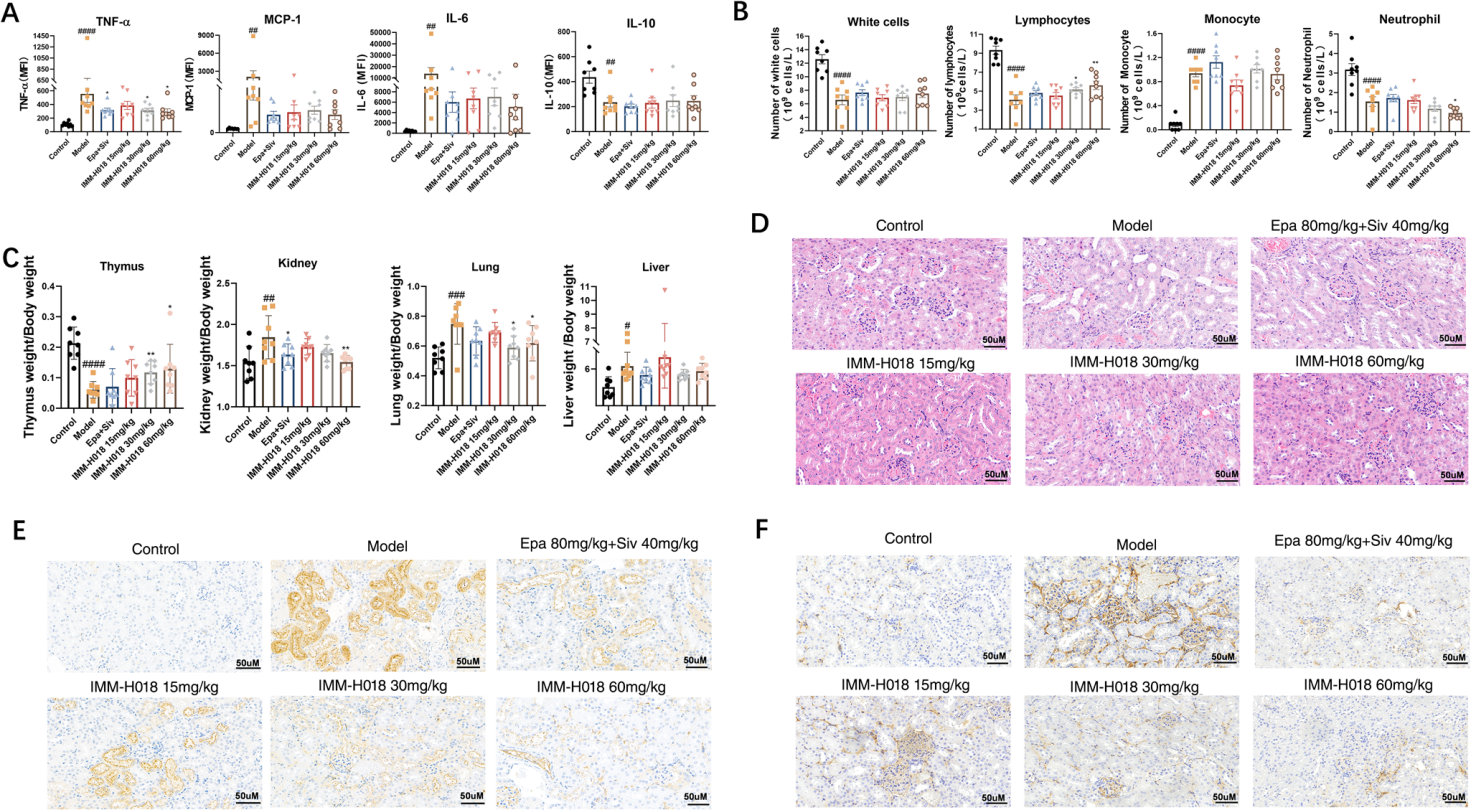
IMM‐H018 ameliorates inflammation, immunosuppression, and AKI in two‐hit sepsis and prevents AKI‐CKD transition. (A, B) Serum concentrations of inflammatory cytokines (TNF‐α, MCP‐1, IL‐6, IL‐10) and white cells, lymphocyte, monocyte and Neutrophil. (C) Relative organ weights (thymus, kidney, lung, liver). (D) Representative HE staining and tubular injury score in kidney tissues. (E) Immunohistochemical staining of NGAL in tubulointerstitium. (F) Immunohistochemical staining of fibronectin in kidney tissues. Data are presented as mean ± SD. *p < 0.05, **p < 0.01 vs. Model group; *^#^p < 0.05, ^##^p < 0.01, ^###^p < 0.001, ^####^p < 0.0001* vs. Control group.

### 3.10 Pharmacokinetic Study

Finally, aims to facilitate the novel drug development, the in vivo pharmacokinetic properties of IMM‐H018 were assessed in mice and rats. As shown in Figure 10A and Table 2, following iv administration, the apparent volume of distribution (V) and clearance (CL) of IMM‐H018 were 307 mL/kg and 44.5 mL/h/kg in mice and 181 mL/kg and 10.5 mL/h/kg in rats, respectively. These results indicated that IMM‐H018 exhibited a relatively limited tissue distribution and slow systemic clearance, with slower clearance in rats than in mice (10.5 vs 44.5 mL/h/kg). After oral administration of IMM‐H018 (15 mg/kg), the compound was absorbed quickly, with plasma concentrations reaching µg/mL levels within 5 min. Absorption was faster in mice than in rats (T_max_: 0.25 h vs 4 h). The C_max_ values were comparable between the two species (38.9 vs 35.3 µg/mL). Compared with mice, rats showed a longer terminal half‐life (t_1/2β_: 13.0 h vs 4.71 h) and higher systemic exposure (AUC_0‐∞_: 728 vs 251 h·µg/mL). The oral bioavailability of IMM‐H018 was 74.4% in mice and 46.7% in rats.

**FIGURE 10:**
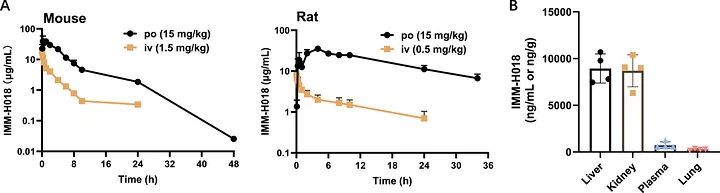
Pharmacokinetics study of IMM‐H018. (A) Pharmacokinetic profiles of IMM‐H018 in mice and rats following oral and intravenous dosing. Data are presented as mean ± SD (*n* = 3). (B) Tissue exposure of IMM‐H018 at 24 h after the final dose in an AKI mouse model (po, 15 mg/kg/day for 3 successive days). Data are presented as mean ± SD (*n* = 4).

**TABLE 2:** Pharmacokinetic parameters of IMM‐H018 in mice and rats.

Parameters	Unit	Mice		Rats	
		Po 15 mg/kg	Iv 1.5 mg/kg	Po 15 mg/kg	Iv 0.5 mg/kg
T_max_	h	0.25	—	4	—
C_max_/C_0_	µg/mL	38.9	20.6	35.3	9.08
t_1/2_	h	4.71	4.78	13.0	12.5
MRT _(0‐t)_	h	7.11	4.76	12.4	7.97
MRT _(0‐∞)_	h	7.15	5.59	19.5	16.2
AUC _(0‐t)_	h·µg/mL	251	32.6	600	38.7
AUC _(0‐∞)_	h·µg/mL	251	33.7	728	52.0
V	mL/kg	—	307	—	181
CL	mL/h/kg	—	44.5	—	10.5
F (%)		**74.4**		**46.7**	

*Note*: Three male mice and rats for each administration route. po formulation: 0.5% CMC. iv formulation: 20%HP‐β‐CD/10% DMSO.

To further characterize target‐tissue exposure of IMM‐H018 in an AKI mouse model, IMM‐H018 concentrations were determined in the kidney (target organ), liver, and lung as representative tissues. Mice received IMM‐H018 by ig at 15 mg/kg/day for 3 successive days. At 24 h post‐final dose, the plasma concentration was 737 ng/mL. Kidney, liver and lung tissue concentrations of IMM‐H018 were 8693 ng/g (T/P = 11.8), 8937 ng/g (T/P = 12.1) and 275 ng/g (T/P = 0.4), respectively. These results suggested that IMM‐H018 was more extensively distributed in the liver and the target organ (kidney), with lower distribution in the lung (Figure 10B).

### 3.11 IMM‐H018 Exhibited Favorable In Vivo Safety Profiles

To preliminarily evaluate the in vivo safety profile of IMM‐H018, a 2‐week repeated‐dose toxicity study was performed in SD rats by oral administration. Body weight changes were monitored throughout the treatment period. As shown in Figure 11A, no obvious treatment‐related body weight loss was observed in either female or male rats following IMM‐H018 administration at doses up to 300 mg/kg. In addition, no gross pathological abnormalities were observed in the major organs at necropsy, indicating the absence of apparent treatment‐related organ toxicity.

**FIGURE 11:**
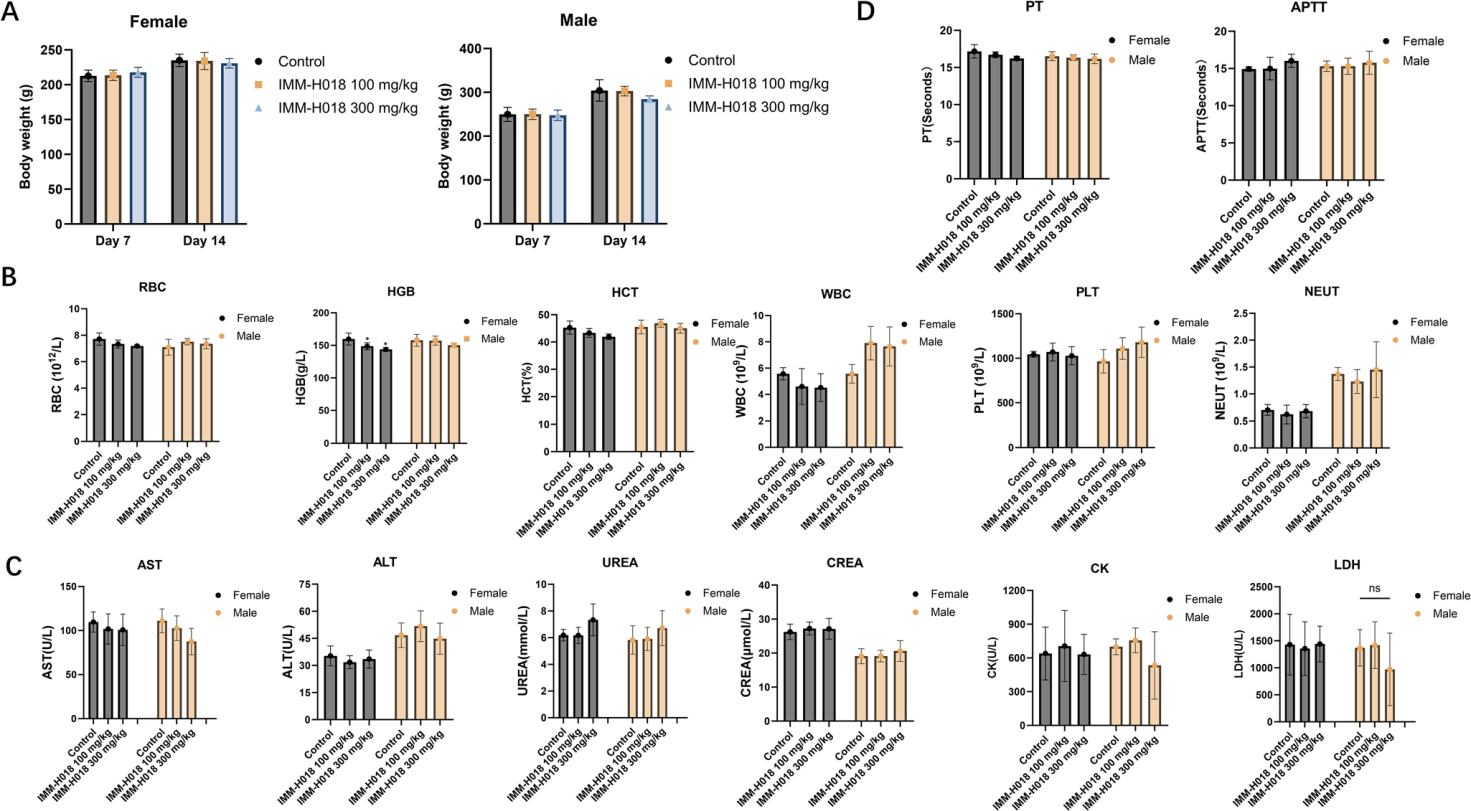
Toxicological evaluation of IMM‐H018 in rats. (A) Body weight changes in female and male SD rats following administration of IMM‐H018 at indicated doses. (B) Hematological parameters (RBC, HGB, HCT, WBC, PLT, and NEUT) in rats after 14 days of treatment. (C) Serum biochemical markers (AST, ALT, UREA, CREA, CK, and LDH) in rats after 14 days of treatment. (D) Coagulation function parameters (PT and APTT) in rats after 14 days of treatment. Data are presented as mean ± SD, with no statistically significant differences between groups (ns, *p* > 0.05).

Hematological analyses demonstrated no significant abnormalities in RBC, WBC, PLT, HCT or NEUT levels after 14 days of repeated IMM‐H018 treatment (Figure 11B). In addition, serum biochemical analyses revealed no obvious alterations in liver function markers (AST and ALT), kidney function indicators (UREA and CREA), or cardiac injury‐related biomarkers (CK and LDH) in 14 days of repeated IMM‐H018‐treated animals compared with the control group (Figure 11C). Considering the critical role of coagulation dysfunction during sepsis progression, coagulation parameters were further evaluated. As shown in Figure 11D, no significant changes in PT or APTT were observed following IMM‐H018 treatment, indicating that IMM‐H018 did not induce obvious coagulation abnormalities under the tested conditions. Detailed statistical results for all serum biochemical, hematological, and urinalysis parameters are summarized in Supplementary Tables S2–S4.

Collectively, these results suggest that IMM‐H018 exhibits favorable in vivo safety and tolerability profiles.

## 4 Discussion

This study reports for the first time the synergistic effect of NE inhibitor combined with IDO1 inhibitor against sepsis and SA‐AKI. We used the commercial NE inhibitor Siv and clinical trial IDO1‐specific inhibitor Epa to prove this hypothesis which derived from several basic research [20, 26, 31, 46], and their mechanism included inhibiting cytokine storm, reversing the T Lymphocyte exhaustion, reducing the renal microthrombosis. In the current study, IDO1 inhibitor did not offset the anti‐inflammation effect of Siv, and as for the golden standard for evaluation of sepsis, survival analysis, we proved that combination had best effect on prolong the life of septic mice. Meanwhile, by using low dosage of LPS challenge, we proved that Epa + Siv could reduce the inflammation, and reverse the immunosuppression by inhibiting the lymphocyte apoptosis in thymus, and reduce the PD‐1 expression in T lymphocytes from blood and spleen. When all the data were collectively analyzed, it became evident that dual inhibition of NE and IDO1 offers superior benefits without antagonistic effects. Specifically, while Siv did not reverse the reduction in lymphocyte count or the elevated expression of immunosuppressive PD‐1, it did not counteract the up‐regulation effects mediated by IDO1 inhibition. Meanwhile, the IDO1 inhibitor did not markedly enhance cytokine production. These findings suggest that this synergized strategy may be effective in treating sepsis. After that, we designed and developed a novel IDO1/NE dual inhibitor IMM‐H018, as a novel drug candidate against sepsis, to normalize the immune dysfunction and restore the renal function during sepsis. Current findings indicated: (1) IMM‐H018 prolongs the survival in CLP septic mice, and protects the renal function in primary and two‐hit septic models; (2) IMM‐H018 reversed the immunosuppression via preventing the T cell apoptosis and enhancing macrophage antigen‐presenting, and reduced the renal microthrombosis and preserves renal tissue perfusion via IDO1‐Kyn‐AhR‐TF axis; (3) IMM‐H018 reduced systemic and renal inflammation via the inhibition of NE activity and NE‐mediated NETs formation. This study offers novel mechanisms underlying the regulation of systemic immune dysfunction and renal perfusion/function in septic injury. All these results not only provide a reasonable treatment strategy for possible clinical intervention, but also provide a potential novel drug candidate for future development against sepsis and SA‐AKI.

Across most efficacy endpoints, IMM‐H018 showed consistently better outcomes than the combination of Epacadostat and Sivelestat, with a subset of kidney injury‐related parameters reaching statistical significance. This overall trend supports the potential advantage of integrating IDO1 and neutrophil elastase inhibition into a single molecular entity for the treatment of sepsis‐associated AKI. Compared with combination therapy, a dual‐target single‐agent strategy may offer several potential advantages. IMM‐H018 provides coordinated pharmacokinetic exposure to both pharmacological activities, avoiding potential discrepancies in the absorption, distribution, metabolism, and elimination of two independently administered agents. In addition, as a dual‐target single agent, IMM‐H018 may provide more coordinated engagement of IDO1 and neutrophil elastase than combination therapy, which depends on the independent pharmacokinetic profiles of two separate drugs. Moreover, IMM‐H018 may simplify dosing and clinical administration. Importantly, repeated‐dose and acute toxicity studies in rodents demonstrated no obvious adverse effects, suggesting a favorable safety profile that could reduce the risk of off‐target or additive toxicities sometimes observed with combination therapy.

Sepsis‐associated morbidity and mortality remain high, partly due to protracted immunosuppression. This condition may arise from impaired pathogen clearance after primary infection or increased susceptibility to secondary infections [46]. The apoptosis of lymphocytes in circulation, thymus and spleen contribute to the long‐lasting immunosuppression in sepsis and correlated with the severity of spies [47]. Previous study has demonstrated that IDO1‐mediated tryptophan metabolites (including Kyn, 3‐Hydroxyanthranilic acid, and quinolinic acid could) promote T cell apoptosis via activation of caspase‐8 and cytochrome c release from mitochondria under inflammation physiopathologic conditions [48], which partially explained the reduction of lymphocyte apoptosis in the present study by IMM‐H018. Blockade of T cell negative co‐stimulatory pathways have been proved to be effective therapeutic approach for primary and secondary sepsis, including PD‐1, PD‐L1 or CTLA‐4 [24], and reversing the PD‐1 mediated immunosuppression and reducing the lymphocyte apoptosis contributes to their major mechanisms. IDO1, also in charge of one of T cell negative co‐stimulatory pathways through metabolic interference, shared the same effect as PD‐1 and CTLA4, and proved to be a potential target for sepsis. In the current studies, IDO1 inhibitor Epa and IMM‐H018 both achieved the similar effect, not only maintaining the lymphocyte number in circulation, spleen and thymus, but also reducing the PD‐1 expression in T cells. Combination of NE inhibitor did not weaken this effect. More interestingly, the IDO1 inhibitor also reduced the peripheral inflammatory cytokine production of TNFα, IL‐6 and IL‐10, in contrast to anti‐PD‐1 antibody, which enhances both the pro‐inflammatory (IL‐6) and immunosuppressive (IL‐10) cytokines [23, 24]. Cytokine storm is another risk factor for application of T cell negative co‐stimulatory inhibitors, and might counteract the net beneficial effect on sepsis. In the current study, both Epa and IMM‐H018 seems have a better safety profile compared with PD‐1 and CTLA4 antibody treatment, and reducing the future risk worry on its usage in clinical trial.

Notably, the clinical development of IDO1 inhibitors in oncology has not been successful despite promising preclinical results, raising important considerations for translational application. Importantly, although IDO1 inhibitors have shown strong therapeutic potential in preclinical tumor models, their clinical translation has been disappointing in oncology. For example, the phase III ECHO‐301 trial evaluating epacadostat combined with pembrolizumab in metastatic melanoma failed to demonstrate improved clinical outcomes over anti‐PD‐1 monotherapy [49]. Several explanations have been proposed, including pathway redundancy (IDO1 vs IDO2/TDO), insufficient patient stratification, compensatory immune escape mechanisms, and the possibility that enzymatic inhibition of IDO1 alone is insufficient to restore anti‐tumor immunity in highly immunosuppressive tumor microenvironments [50, 51]. These translational setbacks highlight the complexity of targeting metabolic immune checkpoints and underscore the importance of disease context when evaluating IDO1 inhibition.

In contrast to cancer, where immune suppression is established through long‐term tumor‐immune co‐evolution and involves redundant inhibitory pathways and stable remodeling of the tumor microenvironment [52], sepsis represents an acute and dynamic immune disorder in which immunosuppression is largely reversible and driven by inflammatory signaling [53]. In this context, IDO1 is rapidly induced by cytokines such as IFN‐γ and functions as a feedback regulator of immune activation through tryptophan metabolism and kynurenine production, suggesting that its inhibition may be more effective in restoring immune homeostasis in sepsis than in chronic tumor settings. In contrast, in cancer, IDO1 inhibition is often insufficient due to pathway redundancy (IDO2/TDO) and compensatory immunosuppressive networks.

In addition to lymphocytes, monocytes contribute to innate immune defense but undergo rapid functional deactivation in sepsis. They show impaired phagocytosis after secondary bacterial challenge [54] and compromised antigen presentation, likely stemming from reduced major histocompatibility complex (MHC) class II expression [55]. As a hallmark of sepsis, decreased MHC II serves as a marker of immunosuppression and correlates with poor clinical outcomes, reflecting a state of global immune dysfunction in critically ill patients. Both inhibition of lymphocytes apoptosis and recovery of macrophage function have been proved to be beneficial for improved the survival of sepsis animals [56], and in the current study, IMM‐H018 administration had achieved both effects. IMM‐H018 reversed the monocyte dysfunction and enhanced the phagosome of macrophages via improving the antigen processing capabilities. We believed that these effects are from the IDO1 inhibition of IMM‐H018.

Most commonly, the LPS‐induced sepsis model was chosen here because features of acute kidney injury (AKI) are more easily observed than in the CLP animal model. Previous studies have also shown that the standard CLP model lacks consistency in reducing reproducible acute kidney or lung injury [57, 58]. Renal tissue hypoperfusion and hypoxia are recognized as major contributors to renal dysfunction during sepsis. In our study, renal hypoperfusion was observed in mice following LPS challenge, as indicated by reduced MSOT signal intensity of total hemoglobin and the presence of thrombosis. These findings are consistent with clinical and large‐animal studies, where reduced renal blood flow (RBF) has been reported in septic patients with kidney injury and in relevant preclinical models. Although NETs contribute to host defense via antimicrobial activity, excessive NETosis during sepsis can trigger intravascular thrombosis, disseminated intravascular coagulation, and multiple organ dysfunction, highlighting its detrimental effects. In the current study, IMM‐H018 significantly reduced NETs formation in the kidneys, meanwhile, reduction of TF production via IDO1‐Kyn‐AhR‐TF axis also contributed to the decreased microthrombosis in the kidneys.

Aims to enhance the drug development, we use the mice and rats, to perform the pharmacokinetics research to investigate the oral absorption and organ distributions. Overall, ADMET Predictor suggested that IMM‐H018 has favorable intestinal permeability, with a predicted effective human jejunal permeability (Peff) of 6.5 × 10^−4^ cm/s, which is consistent with the rapid absorption and moderate‐to‐high oral bioavailability observed in rodents. In addition, ADMET Predictor predicted high plasma protein binding for IMM‐H018 (>95%), consistent with the relatively small volume of distribution of 0.18–0.31 L/kg) and suggesting preferential distribution within the plasma/extracellular compartment with limited tissue distribution. These predictions warrant experimental confirmation. Notably, in an AKI mouse model, IMM‐H018 showed a high T/P ratio (T/P > 10) in the kidney at 24 h after the final dose, supporting adequate exposure in the target organ (kidney) for AKI. The liver, as a major metabolic organ, showed higher exposure, which is reasonable and expected. Considering the elevated exposure in both the kidney and liver, careful safety evaluation of these organs is warranted during further development. Encouragingly, repeated‐dose toxicity studies in rats demonstrated that IMM‐H018 was well tolerated at doses up to 300 mg/kg, without significant effects on body weight, hematological parameters, serum biochemical indicators of hepatic, renal, or cardiac function, or coagulation parameters. Collectively, these findings suggest a favorable preliminary safety profile and indicate that the high renal and hepatic exposure of IMM‐H018 does not translate into detectable organ toxicity under the tested conditions. Nevertheless, comprehensive long‐term toxicological studies will be required to further establish its safety margin and support future clinical development.

The present findings provide preclinical hypothesis‐generating data supporting the concept that IDO1 inhibition may constitute a new therapeutic method for sepsis. Although Epacadostat and other IDO1 inhibitors have shown limited efficacy in inducing remission in cancer patients compared with anti‐PD‐1 or anti‐PD‐1/PD‐L1 strategies aimed at enhancing host immunity [59, 60], sepsis‐another life‐threatening condition arising from host immune dysfunction‐may represent a more suitable indication for this class of drugs. Furthermore, IDO1 inhibitors could be tailored to septic patients with persistently elevated IDO1 enzyme activity in peripheral T cells or monocytes, as reflected by increased plasma kynurenine concentrations. Thus, a readily measurable biomarker could help identify ideal candidates for treatment with IDO1 inhibitors such as IMM‐H018.

There are also several limitations in the current study. First, the in‐depth mechanisms of IMM‐H018 beneficial effect on sepsis and SA‐AKI are still not clear, and needs further study; Second, compared with therapeutic administration, prophylactic dosing of IMM‐H018 appeared to provide more pronounced protective effects. Further studies are warranted to systematically evaluate its efficacy at different treatment time points and to identify biomarkers that could guide early intervention. Therefore, the therapeutic efficacy of IMM‐H018 in a clinically relevant treatment setting requires further validation.

In conclusion, the present study demonstrates that IMM‐H018, a novel dual inhibitor of NE and IDO1, exerts protective effects in septic mice by prolonging survival and enhancing renal perfusion during septic injury. These effects are mediated, at least in part, through the inhibition of the IDO1‐Kyn‐AhR‐TF axis and NE activity. Therefore, IMM‐H018 represents a promising therapeutic agent for primary sepsis, secondary infection‐induced sepsis, and sepsis‐associated kidney injury.

## Author Contributions

**Yi Zhou** and **Xiaodi Zhao** designed the experiments, conducted the experiments, and processed the experimental data. **Xiaochen Pan** was responsible for software operation and resource support. **Zhiling Ma** was responsible for conducting part of the experiments. **Peng Wang** contributed to Project administration. **Hui Wen**, **Huaqing Cui**, **Xiufeng Liao**, and **Kejie Qin** undertook the pharmaceutical chemistry‐related work. **Li Sheng** was in charge of the pharmacokinetic part. **Yuchen Wang** contributed to investigation and methodology. **Sen Zhang** was responsible for the manuscript writing, research supervision, and financial support for the fund.

## Conflicts of Interest

The authors declare no conflicts of interest.

## Supporting information




**Supporting File 1**: advs76504‐sup‐0001‐SuppMat.docx.

**Supporting File 2**: advs76504‐sup‐0002‐Document‐Proof.pptx.

## Data Availability

The data that support the findings of this study are available on request from the corresponding author. The data are not publicly available due to privacy or ethical restrictions.
