# Supplementary material for: A Novel IDO1/NE Dual Inhibitor, IMM‐H018 Prevents the Primary and Secondary Sepsis and Ameliorates the Kidney Injury Through Inhibiting the Cytokine Storm and Microthrombosis, and Reversing Immunosuppression
Source: Adv Sci (Weinh). 2026 Jul 20;13(55):e76504. doi: 10.1002/advs.76504 (PMC13384038; doi:10.1002/advs.76504)
Supplement: Supplementary file 1 [file ADVS-13-e76504-s002.docx]

**Supporting Information**

**Supplemental Figure S1** The gating strategy for apoptosis of T cell in thymus.

**Supplemental Figure S2** The gating strategy for PD-1 expression in CD11b (A) and CD3 cells (B) through flow cytometry.

**Supplemental Figure S3** Determination of percent of PD-1 expression on CD11b^+^ and CD3^+^ cells 24h after LPS challenge (N=6) through flow cytometry. Data are presented as mean ± SD. **P < 0.05* vs. LPS group. *#P < 0.05* vs. Control group.

**Supplemental Figure S4** The gating strategy for PD-1 expression in CD3^+^, CD4^+^ and CD8^+^ lymphocytes from spleen through flow cytometry.

**Supplemental Figure S5** Determination of percent of PD-1 expression on CD3^+^, CD4^+^ and CD8^+^ lymphocytes from spleen 24h after LPS challenge (N=6) through flow cytometry. Data are presented as mean ± SD. **P < 0.05, **P < 0.01, ***P < 0.001* vs. LPS group. *^#^P < 0.05, ^##^P < 0.01* vs. Control group. *^&^P < 0.05, ^&&^P < 0.01*, vs. Epa + Siv group

**Supplemental Figure S6** KEGG analysis by enriched differential expressed mRNAs (LPS vs Control): (A) Upregulated KEGG pathways in kidney tissues by LPS challenge; (B) Down-regulated KEGG pathways in kidney tissues by LPS challenge. Label the mask.

**A**

**B**

**Supplemental Figure S7** Pretreatment with IMM-H018 ameliorates multi-organ injury and pulmonary inflammation in LPS-induced septic mice. (A) Body weight and organ coefficients of the kidney, lung, and liver in each group. (B) Serum levels of the liver injury markers ALT and AST. (C) Protein concentration and neutrophil percentage in bronchoalveolar lavage fluid (BALF). Data are presented as mean ± SD. **P* < 0.05, ***P* < 0.01, ****P* < 0.001 vs. LPS group. *^#^P* < 0.05, *^##^P* < 0.01, *^####^P* < 0.0001 vs. Control group.

**Supplemental Figure S8** Post-treatment with IMM-H018 ameliorates multi-organ injury and pulmonary inflammation in LPS-induced septic mice. (A) Body weight changes and organ coefficients of the thymus, kidney, lung, and liver in each group. (B) Peripheral blood leukocyte differential counts. (C) Serum level of the inflammatory cytokine IL-6. (D) Serum blood urea nitrogen (BUN) levels. (E) Serum levels of the liver injury markers ALT and AST. Data are presented as mean ± SD. **P* < 0.05, ***P* < 0.01 vs. LPS group. *^#^P* < 0.05, *^##^P* < 0.01, *^####^P* < 0.0001 vs. Control group. *^&^P* < 0.05 vs. Epa + Siv group.

**Supplemental Figure S9** Correlation study between serum Kyn/Tyr with BUN in septic mice.

**Supplemental Figure S10** The top 100 differential up or down regulated mRNA (LPS vs control) reversed by IMM-H018 (60mg/kg).

**Supplemental Figure S11** Validation of immunosuppressive status prior to secondary bacterial challenge. Data are presented as mean ± SD. **P < 0.05*, ***P < 0.01*, ****P < 0.001* vs. Control group

**Supplemental Figure S12** The gating strategy for macrophage M1 and M2 subtypes, and MHCI and MHCII.

**A**

**B**

**Supplemental Table S1. Chemical structures of representative IPK-series compounds.**

|   IPK-001 |   IPK-002 |   IPK-003 |
| --- | --- | --- |
|   IPK-004 |   IPK-005 |   IPK-006 |
|   IPK-007 |   IPK-008 |   IPK-009 |
|   IPK-010 |   IPK-011 |   IPK-012 |
|   IPK-013 |   IPK-014 |   IPK-015 |
|   IPK-016 |   IPK-017 |   IPK-018 |
|   IPK-019 |   IPK-020 |   IPK-021 |
|   IPK-022 |   IPK-023 |   IPK-024 |
|   IPK-025 |   IPK-026 |   IPK-027 |
|   IPK-028 |   IPK-029 |   IPK-030 |
|   IPK-031 |   IPK-032 |   IPK-033 |
|   IPK-034 |   IPK-035 |   IPK-036 |
|   IPK-037 |   IPK-038 |   IPK-039 |
|   IPK-040 |   IPK-041 |   IPK-042 |
|   IPK-043 |   IPK-044 |   IPK-045 |
|   IPK-046 |   IPK-047 |   IPK-048 |
|   IPK-049 |   IPK-050 |   IPK-051 |
|   IPK-052 |   IPK-053 |   IPK-054 |
|   IPK-055 |   IPK-056 |   IPK-057 |
|   IPK-058 |   IPK-059 |   IPK-060 |
|   IPK-061 |   IPK-062 |   IPK-063 |
|   IPK-064 |   IPK-065 |   IPK-066 |
|   IPK-067 |   IPK-068 |   IPK-069 |
|  IPK-070 |   IPK-071 |   IPK-072 |
|   IPK-073 |   IPK-074 |   IPK-075 |
|   IPK-076 |   IPK-077 |   IPK-078 |
|   IPK-079 |   IPK-080 |   IPK-081 |
|   IPK-082 |   IPK-083 |   IPK-084 |
|   IPK-085 |   IPK-086 |   IPK-087 |
|   IPK-088 |   IPK-089 |   IPK-090 |
|   IPK-091 |   IPK-092 |   IPK-093 |
|   IPK-094 |   IPK-095 |   IPK-096 |
|   IPK-097 | IPK-098 | IPK-099 |
| IPK-100 | IPK-101 | IPK-102 |
| IPK-103 | IPK-104 | IPK-105 |
| IPK-106 | IPK-107 | IPK-108 |
| IPK-109 | IPK-110 | IPK-111 |
| IPK-112 | IPK-113 | IPK-114 |
| IPK-115 | IPK-116 | IPK-117 |
| IPK-118 | IPK-119 | IPK-120 |
| IPK-121 | IPK-122 | IPK-123 |
| IPK-124 | IPK-125 | IPK-126 |
| IPK-127 | IPK-128 | IPK-129 |
| IPK-130 | IPK-131 | IPK-132 |
| IPK-133 | IPK-134 | IPK-135 |

**Supplemental Table S2. Blood biochemical test in female and male Rats after 14-day repeated oral administration of IMM-H018.**

| Sex | Index | IMM-H018 dosage（mg/kg） | | | |
| --- | --- | --- | --- | --- | --- |
|  |  | 0 | 100 | 300 |  |
| Female | ALT(U/L) | 35.3±5.5 | 31.8±3.6 | 33.5±5.0 |  |
|  | AST(U/L) | 109.7±11.5 | 101.8±17.1 | 100.8±17.9 |  |
|  | TP(g/L) | 63.90±2.95 | 62.68±2.71 | 65.60±3.91 |  |
|  | ALB(g/L) | 40.27±3.25 | 39.40±1.61 | 41.02±2.42 |  |
|  | GLB(g/L) | 23.63±0.49 | 23.28±1.82 | 24.58±3.07 |  |
|  | A/G | 1.73±0.15 | 1.70±0.13 | 1.68±0.23 |  |
|  | TBIL(μmol/L) | 1.073±0.192 | **0.000±0.000*** | **0.003±0.008*** |  |
|  | ALP(U/L) | 154.3±30.0 | 141.0±47.7 | 118.5±36.3 |  |
|  | LDH(U/L) | 1429.3±561.5 | 1355.3±498.0 | 1440.8±331.5 |  |
|  | CK(U/L) | 639.7±234.8 | 705.8±315.8 | 631.7±178.5 |  |
|  | GLU(mmol/L) | 7.593±0.355 | 6.570±0.556 | 6.762±0.568 |  |
|  | UREA(mmol/L) | 6.187±0.435 | 6.168±0.610 | 7.333±1.197 |  |
|  | UA(μmol/L) | 124.53±15.58 | 107.07±11.63 | 100.85±20.56 |  |
|  | CREA(μmol/L) | 26.23±2.27 | 27.23±1.87 | 27.13±3.05 |  |
|  | CHOL(mmol/L) | 1.513±0.106 | **1.745±0.090*** | **2.053±0.414*** |  |
|  | TG(mmol/L) | 0.333±0.068 | 0.313±0.098 | 0.440±0.222 |  |
|  | Ca(mmol/L) | 2.340±0.053 | 2.403±0.055 | **2.483±0.084*** |  |
|  | Na(mmol/L) | 144.0±1.0 | 144.7±0.8 | 144.3±1.2 |  |
|  | K(mmol/L) | 4.13±0.02 | 4.105±0.227 | 4.533±0.448 |  |
|  | Cl(mmol/L) | 107.7±2.5 | 106.2±1.5 | 105.2±1.5 |  |
| Male | ALT(U/L) | 46.7±6.8 | 51.7±8.5 | 44.8±8.6 |  |
|  | AST(U/L) | 111.0±13.5 | 102.7±14 | 87.5±15.1 |  |
|  | TP(g/L) | 55.90±1.10 | **60.38±1.36**** | **60.08±1.69**** |  |
|  | ALB(g/L) | 31.90±0.95 | **35.75±0.60**** | **35.13±1.33**** |  |
|  | GLB(g/L) | 24.00±0.95 | 24.63±1.39 | 24.95±1.53 |  |
|  | A/G | 1.33±0.12 | 1.45±0.10 | 1.40±0.13 |  |
|  | TBIL(μmol/L) | 0.620±0.344 | **0.007±0.008*** | **0.000±0.000*** |  |
|  | ALP(U/L) | 267.7±38.0 | 235.2±34.3 | 197.5±72.3 |  |
|  | LDH(U/L) | 1370.7±339.1 | 1419.5±432.7 | 970.8±671.7 |  |
|  | CK(U/L) | 699.0±70.4 | 756.7±109.9 | 553.3±299.9 |  |
|  | GLU(mmol/L) | 7.390±0.705 | 6.972±1.041 | 7.073±1.668 |  |
|  | UREA(mmol/L) | 5.833±1.064 | 5.908±0.874 | 6.712±1.308 |  |
|  | UA(μmol/L) | 106.50±9.79 | **118.75±5.54*** | 96.7±18.11 |  |
|  | CREA(μmol/L) | 19.07±2.21 | 19.13±1.73 | 20.60±3.06 |  |
|  | CHOL(mmol/L) | 1.393±0.220 | 1.690±0.171 | **1.958±0.168**** |  |
|  | TG(mmol/L) | 0.427±0.272 | 0.383±0.061 | 0.518±0.131 |  |
|  | Ca(mmol/L) | 2.217±0.083 | **2.400±0.048***** | **2.407±0.032***** |  |
|  | Na(mmol/L) | 144.7±1.2 | 143.7±1.2 | 144.2±1.0 |  |
|  | K(mmol/L) | 4.573±0.216 | 4.837±0.121 | 4.768±0.305 |  |
|  | Cl(mmol/L) | 108.0±1.0 | **105.8±1.3*** | **104.5±1.0**** |  |

Data was represented by mean ± standard deviation. Compared with vehicle control, **P*≤0.05, ***P*≤0.01, ****P*≤0.001

ALT: Alanine Aminotransferase

AST: Aspartate Aminotransferase

TP: Total Protein

ALB: Albumin

GLB: Globulin

A/G: Albumin/Globulin Ratio

TBIL: Total Bilirubin

ALP: Alkaline Phosphatase

LDH: Lactate Dehydrogenase

CK: Creatine Kinase

GLU: Glucose

UREA: Urea

UA: Uric Acid

CREA: Creatinine

CHOL: Total Cholesterol

TG: Triglycerides

Ca: Calcium

Na: Sodium

K: Potassium

Cl: Chloride

**Supplemental Table S3. Hematological analysis in female and male Rats after 14-day repeated oral administration of IMM-H018.**

| Sex | Index | IMM-H018 dosage（mg/kg） | | | |
| --- | --- | --- | --- | --- | --- |
|  |  | 0 | 100 | 300 |  |
| Female | RBC (10^12/L) | 7.717±0.465 | 7.337±0.300 | 7.190±0.066 |  |
|  | HGB (g/L) | 159.7±9.2 | **148.7±5.4*** | **143.7±2.8*** |  |
|  | HCT (%) | 45.27±2.40 | 43.33±1.61 | 41.95±1.04 |  |
|  | MCV (fL) | 58.70±1.80 | 59.12±1.34 | 58.35±1.47 |  |
|  | MCH (pg) | 20.73±0.61 | 20.25±0.44 | 19.97±0.45 |  |
|  | MCHC (g/L) | 352.7±1.5 | **342.8±3.8**** | **342.5±2.8**** |  |
|  | #Reti (10^9/L) | 205.80±14.79 | 238.62±30.31 | 199.55±50.66 |  |
|  | %Reti (%) | 2.673±0.225 | 3.258±0.435 | 2.777±0.711 |  |
|  | WBC (10^9/L) | 5.57±0.47 | 4.60±1.36 | 4.52±1.06 |  |
|  | #NEUT (10^9/L) | 0.70±0.10 | 0.62±0.17 | 0.68±0.12 |  |
|  | #LYMPH (10^9/L) | 4.70±0.52 | 3.82±1.28 | 3.73±1.08 |  |
|  | #MONO (10^9/L) | 0.10±0.00 | 0.07±0.05 | 0.03±0.05 |  |
|  | #EOS (10^9/L) | 0.07±0.06 | 0.04±0.05 | 0.05±0.05 |  |
|  | #BASO (10^9/L) | 0.00±0.00 | 0.00±0.00 | 0.00±0.00 |  |
|  | %NEUT (%) | 12.33±1.96 | 13.75±3.07 | 15.95±5.43 |  |
|  | %LYMPH (%) | 84.20±2.17 | 82.97±3.50 | 81.18±5.49 |  |
|  | %MONO (%) | 1.50±0.10 | 1.28±0.40 | 1.05±0.23 |  |
|  | %EOS (%) | 1.07±0.40 | 1.02±0.34 | 1.05±0.47 |  |
|  | %BASO (%) | 0.10±0.00 | 0.17±0.08 | 0.13±0.08 |  |
|  | PLT (10^9/L) | 1044.0±33.8 | 1070.5±100.7 | 1028.2±101.0 |  |
|  | MPV (fL) | 7.57±0.75 | 8.03±0.30 | 7.98±0.35 |  |
| Male | RBC (10^12/L) | 7.097±0.601 | 7.515±0.234 | 7.353±0.374 |  |
|  | HGB (g/L) | 157.7±9.1 | 157.3±6.9 | 150.3±3.5 |  |
|  | HCT (%) | 45.50±2.52 | 46.87±1.45 | 45.02±1.76 |  |
|  | MCV (fL) | 64.20±2.25 | 62.37±1.41 | 61.32±2.83 |  |
|  | MCH (pg) | 22.27±1.19 | 20.93±0.93 | **20.48±0.63*** |  |
|  | MCHC (g/L) | 346.3±7.6 | 335.5±8.2 | 334.3±9.2 |  |
|  | #Reti (10^9/L) | 239.57±43.13 | 302.88±53.73 | 283.50±59.80 |  |
|  | %Reti (%) | 3.400±0.765 | 4.028±0.658 | 3.878±0.909 |  |
|  | WBC (10^9/L) | 5.57±0.71 | 7.90±1.27 | 7.65±1.49 |  |
|  | #EUTC (10^9/L) | 1.37±0.12 | 1.23±0.22 | 1.45±0.52 |  |
|  | #LYMPH (10^9/L) | 3.93±0.80 | **6.33±1.18*** | **5.95±1.03*** |  |
|  | #MONO (10^9/L) | 0.13±0.06 | 0.18±0.08 | 0.12±0.04 |  |
|  | #EOS (10^9/L) | 0.00±0.00 | 0.05±0.05 | 0.03±0.05 |  |
|  | #BASO (10^9/L) | 0.00±0.00 | 0.00±0.00 | 0.00±0.00 |  |
|  | %NEUT (%) | 25.10±3.51 | **15.95±3.42*** | 18.18±4.83 |  |
|  | %LYMPH (%) | 70.23±5.05 | **79.95±3.53*** | **78.35±4.80*** |  |
|  | %MONO (%) | 2.37±0.86 | 2.07±0.95 | 1.65±0.37 |  |
|  | %EOS (%) | 0.63±0.35 | 0.65±0.14 | 0.57±0.31 |  |
|  | %BASO (%) | 0.13±0.06 | 0.12±0.08 | 0.12±0.04 |  |
|  | PLT (10^9/L) | 965.7±130.9 | 1109.8±121.5 | 1179.8±170.3 |  |
|  | MPV (fL) | 7.43±0.86 | 7.70±0.66 | 7.98±0.31 |  |

Data was represented by mean ± standard deviation. Compared with vehicle control, **P*≤0.05, ***P*≤0.01

RBC: Red Blood Cell Count

HGB: Hemoglobin

HCT: Hematocrit

MCV: Mean Corpuscular Volume

MCH: Mean Corpuscular Hemoglobin

MCHC: Mean Corpuscular Hemoglobin Concentration

#Reti: Reticulocyte Count

%Reti: Reticulocyte Percentage

WBC: White Blood Cell Count

#NEUT: Absolute Neutrophil Count

#LYMPH: Absolute Lymphocyte Count

#MONO: Absolute Monocyte Count

#EOS: Absolute Eosinophil Count

#BASO: Absolute Basophil Count

%NEUT: Neutrophil Percentage

%LYMPH: Lymphocyte Percentage

%MONO: Monocyte Percentage

%EOS: Eosinophil Percentage

%BASO: Basophil Percentage

PLT: Platelet Count

MPV: Mean Platelet Volume

**Supplemental Table S4. Urine biochemical test in female and male Rats after 14-day repeated oral administration of IMM-H018.**

| Sex | | Index | | IMM-H018 dosage（mg/kg） | | |  |  |
| --- | --- | --- | --- | --- | --- | --- | --- | --- |
|  |  |  |  | 0 | 100 | 300 | |  |
| Female | | SG 1.005 | | 2/3 | 3/6 | 4/6 | |  |
|  |  | 1.010 | | 0/3 | 3/6 | 1/6 | |  |
|  |  | 1.015 | | 1/3 | 0/6 | 1/6 | |  |
|  |  | LEU | |  |  |  | |  |
|  |  | neg | | 3/3 | 6/6 | 6/6 | |  |
|  |  | NIT | |  |  |  | |  |
|  |  | neg | | 1/3 | 3/6 | 5/6 | |  |
|  |  | + | | 2/3 | 3/6 | 1/6 | |  |
|  |  | pH | |  |  |  | |  |
|  |  | 5 | | 0/3 | 1/6 | 1/6 | |  |
|  |  | 6 | | 1/3 | 4/6 | 3/6 | |  |
|  |  | 7 | | 2/3 | 1/6 | 1/6 | |  |
|  |  | 8 | | 0/3 | 0/6 | 1/6 | |  |
|  |  | ERY | |  |  |  | |  |
|  |  | neg | | 3/3 | 6/6 | 6/6 | |  |
|  |  | PRO | |  |  |  | |  |
|  |  | neg | | 3/3 | 6/6 | 6/6 | |  |
|  |  | GLU | |  |  |  | |  |
|  |  | norm | | 3/3 | 6/6 | 6/6 | |  |
|  |  | ASC | |  |  |  | |  |
|  |  | neg | | 3/3 | 5/6 | 0/6 | |  |
|  |  | ± | | 0/3 | 1/6 | 5/6 | |  |
|  |  | + | | 0/3 | 0/6 | 1/6 | |  |
|  |  | KET | |  |  |  | |  |
|  |  | neg | | 3/3 | 6/6 | 6/6 | |  |
|  |  | UBG | |  |  |  | |  |
|  |  | norm | | 3/3 | 6/6 | 6/6 | |  |
|  |  | BIL | |  |  |  | |  |
|  |  | neg | | 3/3 | 6/6 | 6/6 | |  |
| Male | SG 1.005 | | 2/3 | | 4/6 | 2/6 | |  |
|  | 1.010 | | 1/3 | | 2/6 | 2/6 | |  |
|  | 1.015 | | 0/3 | | 0/6 | 2/6 | |  |
|  | LEU | |  | |  |  | |  |
|  | neg | | 3/3 | | 6/6 | 5/6 | |  |
|  | ± | | 0/3 | | 0/6 | 1/6 | |  |
|  | NIT | |  | |  |  | |  |
|  | neg | | 3/3 | | 3/6 | 5/6 | |  |
|  | + | | 0/3 | | 3/6 | 1/6 | |  |
|  | pH | |  | |  |  | |  |
|  | 6 | | 0/3 | | 2/6 | 3/6 | |  |
|  | 7 | | 1/3 | | 1/6 | 2/6 | |  |
|  | 8 | | 2/3 | | 3/6 | 1/6 | |  |
|  | ERY | |  | |  |  | |  |
|  | neg | | 3/3 | | 6/6 | 6/6 | |  |
|  | PRO | |  | |  |  | |  |
|  | neg | | 3/3 | | 6/6 | 6/6 | |  |
|  | GLU | |  | |  |  | |  |
|  | norm | | 3/3 | | 6/6 | 6/6 | |  |
|  | ASC | |  | |  |  | |  |
|  | neg | | 3/3 | | 0/6 | 0/6 | |  |
|  | ± | | 0/3 | | 6/6 | 4/6 | |  |
|  | + | | 0/3 | | 0/6 | 2/6 | |  |
|  | KET | |  | |  |  | |  |
|  | neg | | 3/3 | | 6/6 | 5/6 | |  |
|  | + | | 0/3 | | 0/6 | 1/6 | |  |
|  | UBG | |  | |  |  | |  |
|  | norm | | 3/3 | | 6/6 | 6/6 | |  |
|  | BIL | |  | |  |  | |  |
|  | neg | | 3/3 | | 6/6 | 6/6 | |  |

SG: Specific Gravity

LEU: Leukocytes

NIT: Nitrite

pH: Urine pH

ERY: Erythrocytes (Occult Blood/Red Blood Cells)

PRO: Protein

GLU: Glucose

ASC: Ascorbic Acid (Vitamin C)

KET: Ketones

UBG: Urobilinogen

BIL: Bilirubin
