# Supplementary figures and images for: A Novel IDO1/NE Dual Inhibitor, IMM‐H018 Prevents the Primary and Secondary Sepsis and Ameliorates the Kidney Injury Through Inhibiting the Cytokine Storm and Microthrombosis, and Reversing Immunosuppression
Source: Adv Sci (Weinh). 2026 Jul 20:e76504. Online ahead of print. doi: 10.1002/advs.76504 (PMC13384038; doi:10.1002/advs.76504)

## Slide 1
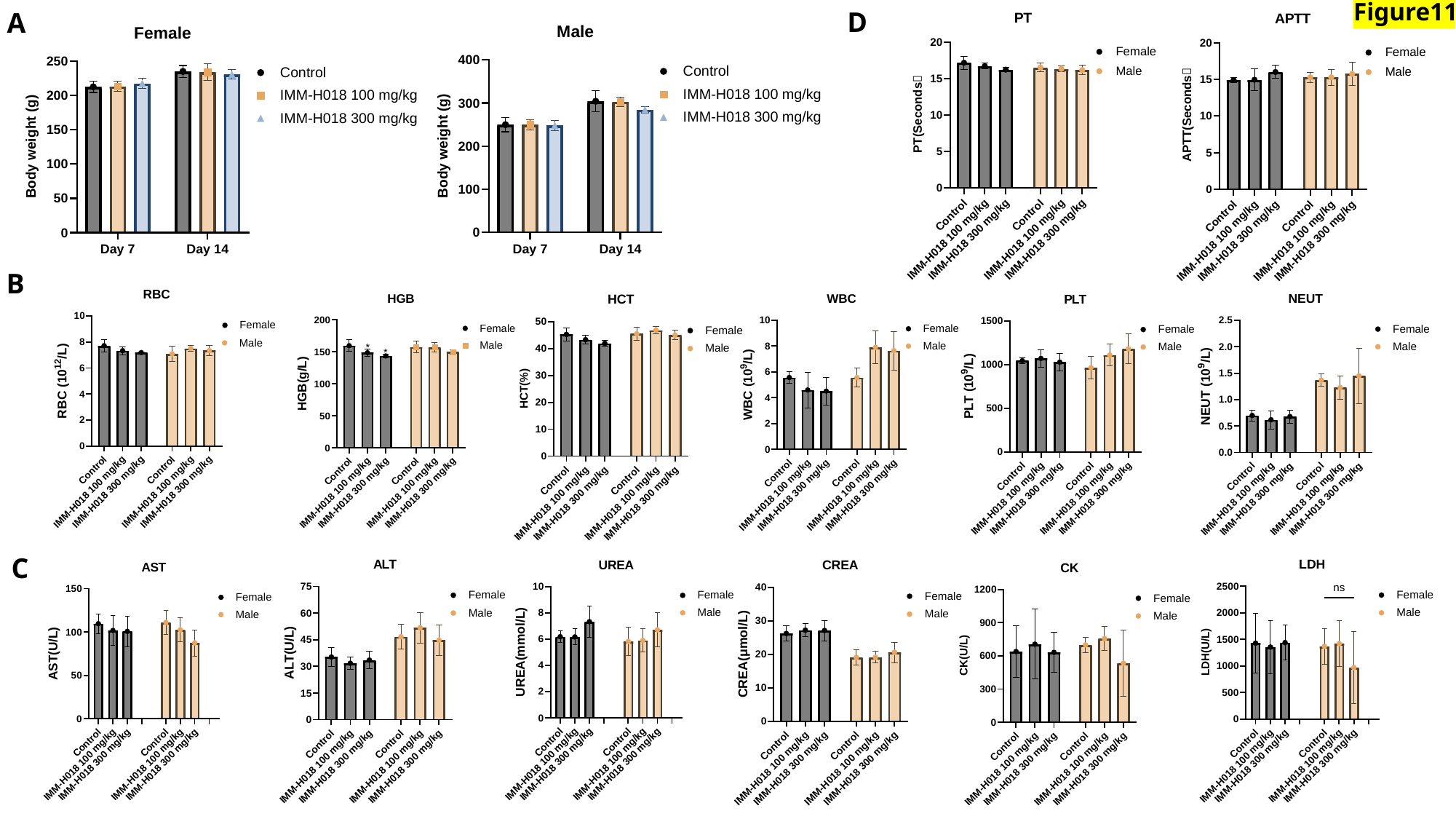

D
Figure11
A
B
C

## Slide 2
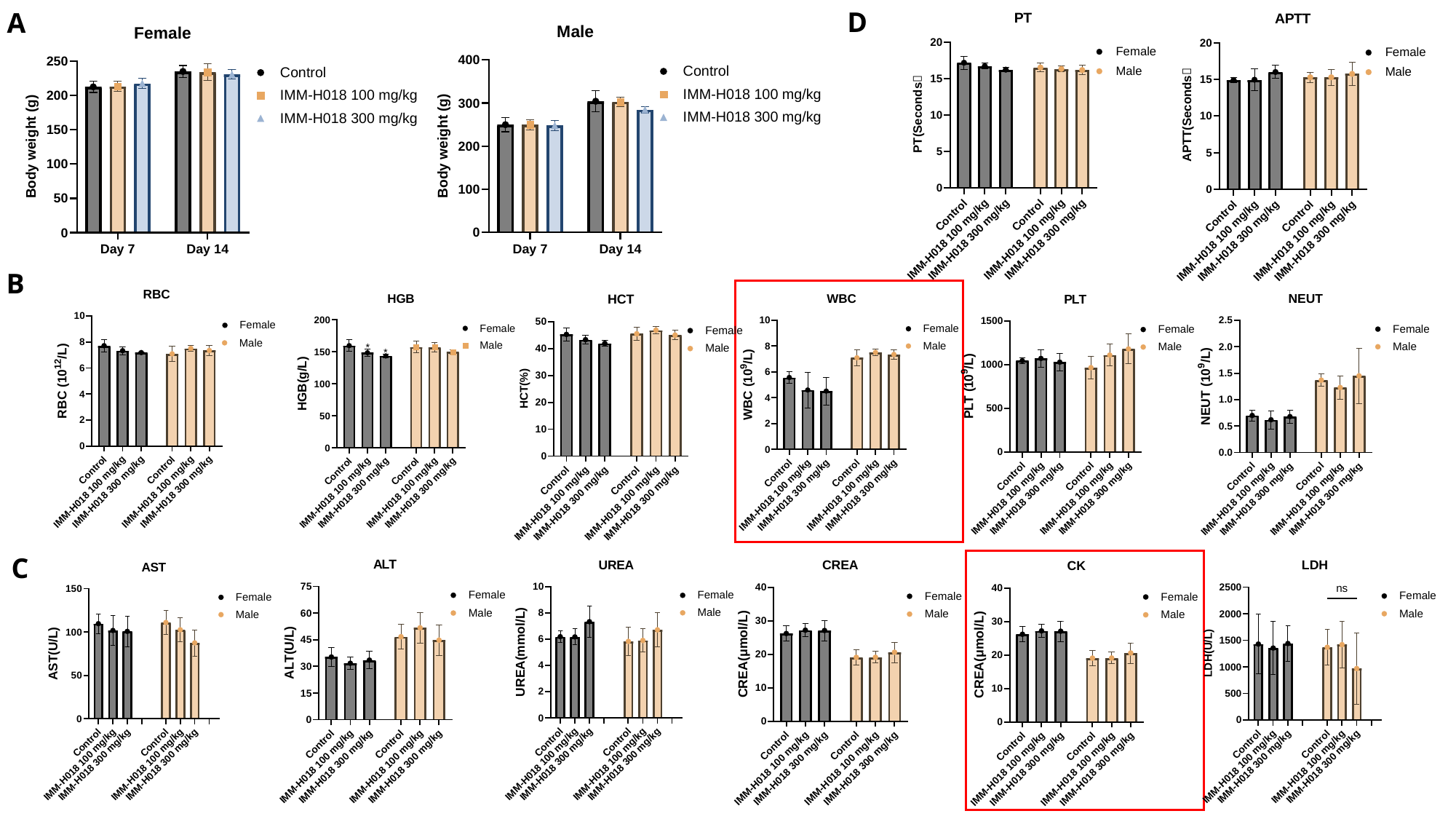

D
A
B
C

Supplement: Supplementary file 2 — Supporting File 2: advs76504‐sup‐0002‐Document‐Proof.pptx. [file ADVS-9999-e76504-s002.pptx]
